# A bacteria-releasing mucoadhesive sheet for localized delivery and penetrative therapy of gastrointestinal cancer

**DOI:** 10.1016/j.mtbio.2026.103710

**Published:** 2026-09-23

**Authors:** Jihun Lee, Sana Ashraf, Eunji Kim, Hyo-Jin Lee, Joowon Park, Heung Jin Jeon, SeungBeum Suh, Sukho Park

**Affiliations:** a Department of Robotics Engineering, Daegu Gyeongbuk Institute of Science and Technology (DGIST), Daegu, 42988, Republic of Korea; b Bionics Research Center, Korea Institute of Science and Technology (KIST), Seoul, 02792, Republic of Korea; c Division of Bio-Medical Science & Technology, University of Science and Technology (UST), Daejeon, 34113, Republic of Korea; d Department of Medical Science, College of Medicine, Chungnam National University, Daejeon, 35015, Republic of Korea; e School of Electrical and Electronic Engineering, University of Ulsan, Ulsan, 44610, Republic of Korea; f Cancer Research Institute, Chungnam National University, Daejeon, 34134, Republic of Korea

**Keywords:** Therapeutic bacterial delivery, Gastrointestinal cancer therapy, Engineered bacteria, Localized tumor treatment

## Abstract

Bacteria-based cancer therapy shows promise for solid tumors, but effective localization and long-term retention in the gastrointestinal (GI) tract remain limited by peristalsis and fluid flow. We developed a Bacteria-Releasing Mucoadhesive Sheet (BRMS), a deployable multilayered platform for precise localized, active bacterial delivery. The BRMS consists of three functional layers: an unrolling layer for rapid deployment and conformal adhesion to the mucosa; a guard layer to minimize bacterial loss and enforce unidirectional release; and a therapeutic layer loaded with engineered *Salmonella* secreting the anticancer protein VC1. In its rolled state, the BRMS is compatible with delivery via standard endoscopes or capsule robots. Upon deployment, it protects bacteria from the luminal environment, prolongs local retention, and promotes penetration into deep tumor tissue. We validated its spatiotemporally controlled delivery and deployment through *ex vivo* experiments. In a CT-26 colon cancer mouse model, the BRMS achieved robust tumor growth inhibition and regression without detectable systemic toxicity. In conclusion, the BRMS is a novel bacterial delivery platform that overcomes GI physiological barriers, enabling sustained bacterial infiltration into deep tumor regions, and offering a powerful strategy to extend the therapeutic potential of bacteria-based cancer therapies.

## Highlights

• Development of a Bacteria-Releasing Mucoadhesive Sheet (BRMS) for GI cancer therapy.
• Multilayered design ensures conformal adhesion and unidirectional bacterial release.
• Compatible with endoscopic and magnetically actuated capsule robot delivery systems.
• Engineered Salmonella enables active deep tumor penetration and localized therapy.
• Robust tumor regression achieved *in vivo* with excellent systemic biosafety.

## Graphical abstract

This study introduces a biohybrid drug delivery platform, BRMS (Bacteria-releasing Mucoadhesive Sheet), designed to overcome gastrointestinal (GI) flow and achieve deep tumor penetration. BRMS features a novel multi-layered structure hosting living engineered *Salmonella*. The platform prevents rapid washout by GI flow via robust mucoadhesion and utilizes endoscopic or capsule device deployment compatible with actual clinical procedures. Crucially, upon deployment, the bacteria actively invade deep into the tumor mass via intrinsic motility and, upon induction, continuously secrete the anticancer protein VC1 locally. The BRMS system achieves effective tumor regression validated through *in vivo* studies, paving a new way for target-specific GI cancer therapies.

**Figure:**
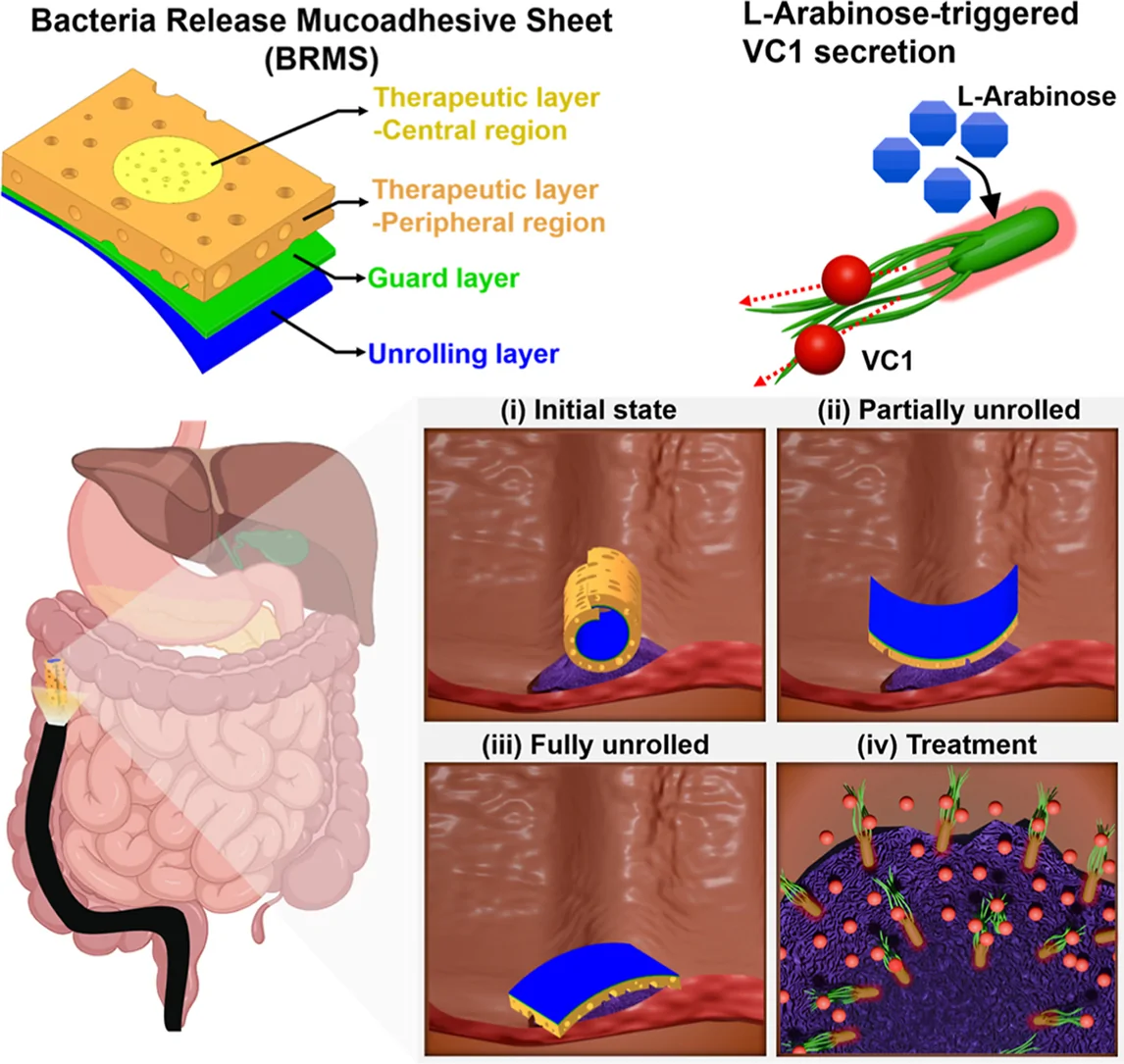

## 1 Introduction

Gastrointestinal (GI) cancer is a significant global health burden characterized by high incidence and mortality [1,2]. Currently, surgical resection and systemic chemotherapy are considered the standard of care [3]. However, surgical resection is highly invasive and often associated with substantial postoperative recurrence rates [4]. Similarly, systemic chemotherapy frequently induces dose-limiting systemic toxicity and serious adverse side effects, significantly compromising patient quality of life [5]. To address the limitations of conventional therapies, localized treatment strategies utilizing hydrogels and patch-based drug delivery systems are being actively investigated for solid tumors within the GI tract [6,7]. Nevertheless, these approaches face a critical limitation: they primarily deliver drugs to the tumor surface and fail to overcome the mechanical and biological barriers of the dense and rigid tumor microenvironment (TME), thereby limiting effective penetration into deep tumor tissue [8].

To overcome the limited intratumoral penetration of conventional therapeutics, active drug delivery strategies that utilize externally applied or self-generated forces have recently gained growing attention. A representative example is a microneedle patch loaded with magnesium (Mg) particles, which exploits the propulsive force generated by hydrogen (H_2_) bubbles formed when Mg reacts with physiological fluids, thereby actively driving drugs deep into cutaneous tissue [9]. Although this approach has demonstrated improved therapeutic efficacy, it has largely been investigated for transdermal drug delivery applications. As an alternative strategy that overcomes these constraints, recent studies have focused on leveraging the inherent motility and tumor-targeting capabilities of living microorganisms, such as bacteria and algae [10,11]. Certain bacterial species preferentially accumulate and proliferate within immunosuppressive and hypoxic tumor microenvironments, where reduced immune surveillance enables selective bacterial colonization. This intrinsic tumor tropism, rather than motility alone, underlies their potential for deep intratumoral targeting [12,13]. Despite this well-established tumor tropism, delivering bacteria to GI tumors in a clinically effective manner remains challenging. Various approaches, such as intravenous administration [14] and direct local injection of bacterial suspensions [15], have been explored, but they commonly suffer from insufficient retention of bacteria at the target lesion—whether due to systemic clearance by host immunity or rapid wash-out by peristalsis, mucus turnover, and continuous luminal fluid flow—such that only a small fraction of the administered bacteria persists long enough to colonize and penetrate the tumor. In the dynamic GI environment in particular, this poor local retention constitutes the rate-limiting factor governing how effectively the intrinsic motility and tumor tropism of bacteria can be translated into deep intratumoral penetration and a sustained, localized therapeutic effect.

In this study, we present a Bacteria-Releasing Mucoadhesive Sheet (BRMS) designed to overcome physiological barriers of the GI tract and enable precise delivery of therapeutic bacteria into deep tumor tissues. The BRMS directly addresses this retention–penetration bottleneck. Through the strong mucoadhesion of its therapeutic layer, the BRMS firmly anchors to the mucosal surface and resists the shear forces of peristalsis and luminal fluid flow, thereby markedly prolonging the local retention of the loaded *Salmonella* at the target lesion. This sustained retention establishes a stable, high-concentration bacterial reservoir directly at the lesion, which in turn provides the released, motile *Salmonella* with sufficient time and local density to actively penetrate the overlying mucus barrier and infiltrate deep into the tumor tissue. Ultimately, the enhanced retention afforded by the BRMS directly translates into improved deep intratumoral penetration of *Salmonella*. In addition, the BRMS is designed to address the three major barriers to GI drug delivery: the gastric acid barrier is avoided because the BRMS is delivered directly to the target lesion via an endoscope or a magnetically actuated capsule robot—rather than administered as a passively swallowed formulation—and remains sealed within the delivery device until deployment, thereby minimizing exposure to the acidic gastric environment; the mucus barrier is overcome through mucoadhesion combined with the active penetration of motile *Salmonella*; and the epithelial tight-junction barrier is circumvented by the locoregional mode of action, in which the released bacteria act directly at the tumor site without requiring systemic paracellular absorption. The BRMS is designed for deployment in a rolled configuration via endoscopic tools or capsule robots. As shown in Fig. 1a, the BRMS features a multilayered functional architecture to enhance mechanical stability and therapeutic efficiency. First, the unrolling layer swells upon exposure to intestinal fluids, allowing the rolled BRMS to rapidly unfurl and adhere conformally to curved mucosal surfaces. The guard layer effectively blocks the leakage of therapeutic bacteria into the GI lumen, ensuring unidirectional bacterial release toward the tumor tissue. Finally, the therapeutic layer is spatially divided into a central and a peripheral region. The central region acts as a reservoir for bacterial loading and release, while the surrounding peripheral region provides a strong mucoadhesive force, firmly anchoring the BRMS to the target lesion even under vigorous peristalsis. Fig. 1b illustrates the VC1-secreting *Salmonella* loaded within the central region of the therapeutic layer. This genetically engineered *Salmonella* is inducible by L-arabinose to secrete the anticancer protein VC1, thereby inducing cancer cell death. The on-site activation mechanism within the therapeutic layer is depicted in Fig. 1c. Initially, inactive VC1-secreting *Salmonella* and L-arabinose are co-encapsulated within the central region. Upon hydration and deployment of the BRMS, the *Salmonella* is released and simultaneously exposed to the co-loaded L-arabinose, thereby triggering bacterial activation and subsequent VC1 secretion.

**Fig. 1:**
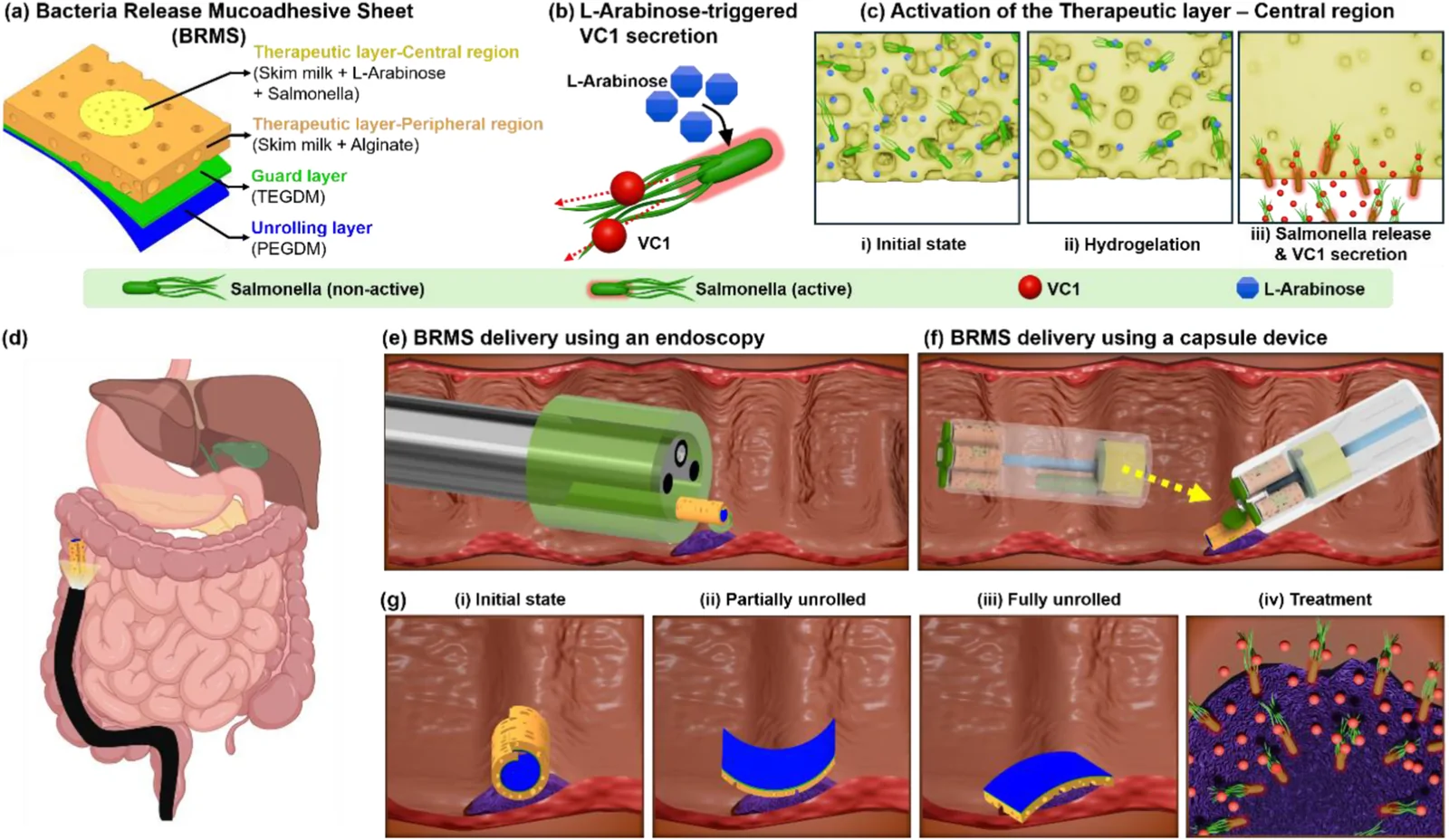
Schematic illustration of Bacteria-Releasing Mucoadhesive Sheet (BRMS) for targeted GI cancer therapy. (a) Schematic representation of the multilayered BRMS architecture, consisting of an unrolling layer (PEGDM), a guard layer (TEGDM), and a therapeutic layer composed of a mucoadhesive peripheral region (alginate/skim milk) and a bacteria-loaded central region (skim milk/VC1-secreting *Salmonella*). (b) Illustration of the mechanism of action, showing L-arabinose-induced VC1 secretion by genetically engineered *Salmonella*. (c) Stepwise activation process of the central region of the therapeutic layer: (i) the freeze-dried initial state, (ii) hydrogel formation and bacterial activation upon absorption of intestinal fluid, and (iii) simultaneous release of *Salmonella* and VC1 secretion. (d) Schematic depiction of the targeted deployment of the BRMS within the GI tract. (e, f) Endoscope-assisted and magnetically actuated capsule robot-based delivery of the BRMS. (g) Therapeutic sequence of the BRMS action on a GI tumor: (i-iii) fluid-triggered self-unrolling and conformal adhesion to the tumor surface, followed by (iv) active penetration of VC1-secreting *Salmonella* into the tumor core for localized therapy.

As illustrated in Fig. 1d, the proposed BRMS is engineered for precise, localized therapy within the GI tract. The platform can be deployed via the working channel of an endoscope (Fig. 1e) or by a magnetically actuated capsule robot (Fig. 1f). Notably, these medical devices enable the sequential deployment of multiple BRMS units, facilitating the simultaneous treatment of multiple lesions in a single procedure. The overall workflow for treating GI solid tumors using the BRMS is depicted in Fig. 1g. Specifically, the process involves the following steps: (i) the BRMS is delivered to the target site in a rolled configuration; (ii) it gradually unrolls upon absorbing intestinal fluids; (iii) it adheres conformally and stably to the curved mucosal surface; and (iv) securely anchored by strong mucoadhesion, the sheet releases VC1-secreting *Salmonella*. These bacteria then actively penetrate deep tumor tissue and continuously secrete the anticancer protein VC1, thereby eliciting a therapeutic effect against GI cancer. VC1, also known as α-conotoxin Vc1.1, is a 16-amino-acid peptide originally identified from the marine cone snail *Conus victoriae* and is a well-characterized antagonist of the α9α10 nicotinic acetylcholine receptor (α9α10 nAChR) [[16], [17], [18]]. α9-containing nAChRs are expressed in several cancer types and have been implicated in tumor-associated processes, including cell proliferation, survival, and migration [16,19]. Accordingly, pharmacological inhibition of α9-containing nAChRs by α-conotoxins such as Vc1.1 has been reported to suppress cancer cell proliferation, providing a mechanistic rationale for its investigation as a potential anticancer peptide [20].

In this study, we developed and systemically characterized a BRMS for the treatment of GI solid tumors and investigated the tumor-penetrating capability of *Salmonella* released from the BRMS. Furthermore, we assessed the cancer cell-killing efficacy of the BRMS through *in vitro* cytotoxicity assays and demonstrated the feasibility of its precise deployment within a physiologically relevant GI environment via *ex vivo* experiments. Finally, we established the therapeutic efficacy and highlighted the potential clinical applicability of this BRMS-based bacterial delivery strategy through *in vivo* anticancer efficacy evaluations.

## 2 Experimental section

### 2.1 Design of BRMS

To overcome the complex physical, chemical, and hydrodynamic challenges within the GI tract and enhance localized therapeutic efficacy, we developed a functional, multilayered BRMS by integrating biocompatible polymeric materials with genetically engineered *Salmonella*. In designing the BRMS, we specifically considered the three major barriers that hinder GI drug delivery—the acidic gastric environment, the mucus barrier, and the epithelial tight-junction barrier—and rationally engineered the platform to address each of them. First, unlike conventional oral formulations that are swallowed and exposed to gastric acid while passing through the stomach, the BRMS is delivered directly to the target lesion in a dry, rolled state using an endoscope or a magnetically actuated capsule robot. Until it reaches the target site, the BRMS is kept within the working channel of the endoscope or the sealed storage compartment of the capsule robot, and the distal Ecoflex cap or the Ecoflex lids assembly blocks the ingress of luminal fluids (Figs. S2 and S4); the BRMS and its encapsulated bacteria therefore remain protected from direct contact with gastric acid and digestive fluids while traversing the upper GI tract. Second, to overcome the mucus barrier that limits the retention and penetration of therapeutic agents, the mucoadhesive peripheral region of the therapeutic layer firmly anchors the BRMS to the mucosal surface against peristalsis and luminal fluid flow, while the guard layer enforces unidirectional bacterial release toward the tumor, and the released *Salmonella* actively penetrate the mucus layer through their intrinsic flagellar motility. Third, whereas conventional oral therapeutics must cross the epithelial tight junctions to reach the systemic circulation, the BRMS instead exerts its therapeutic effect locoregionally at the tumor site: the motile *Salmonella* actively invade the tumor tissue and secrete the anticancer protein VC1 *in situ*, thereby circumventing the need for paracellular transport across tight junctions. The BRMS was rationally engineered to simultaneously ensure structural stability during delivery, precise and robust adhesion to the target site, and active intratumoral penetration of therapeutic agents.

As illustrated in Fig. 1a, the BRMS is constructed from three functional layers. First, the unrolling layer is fabricated from PEGDM, which exhibits high swelling capacity upon water absorption [21]; this property enables the BRMS, delivered in a rolled state, to autonomously unroll upon contact with intestinal fluids. This mechanism allows the BRMS to adhere conformally to the variously curved structures within the GI tract. Second, the guard layer is composed of TEGDM, which possesses near-zero permeability, thereby effectively preventing the diffusion of VC1-secreting *Salmonella* into the lumen and ensuring unidirectional release toward the tumor [22]. In particular, PEGDM and TEGDM are well established as bio-inert and non-cytotoxic materials, allowing them to function safely *in vivo* without inducing inflammatory responses or tissue damage [23]. Third, the therapeutic layer, which directly interfaces with the lesion, is spatially divided into central and peripheral regions for distinct functional roles. The central region contains skim milk, genetically engineered VC1-secreting *Salmonella*, and L-arabinose to activate bacterial function, playing a critical role in therapeutic release after the BRMS adheres to the target site. Here, skim milk serves as a model cryoprotectant that maintains bacterial viability by preventing cell membrane damage caused by ice-crystal formation during the lyophilization process [24]. The peripheral region of the therapeutic layer consists of a mucoadhesive composite of alginate and skim milk. The abundant carboxyl and hydroxyl groups in alginate form strong hydrogen-bonding interactions with mucin in the GI mucosa [25]. This mucoadhesion enables the BRMS to resist shear stresses caused by peristalsis and continuous fluid flow, acting as an anchoring layer that prevents wash-out and ensures stable, long-term retention at the target site.

The enhanced therapeutic efficacy of the BRMS is primarily attributed to the active intratumoral penetration of the loaded genetically engineered, attenuated VC1-secreting *Salmonella*. The *Salmonella* strain used in this study was engineered to secrete the therapeutic peptide VC1 via the flagellar type III secretion system (fT3SS). As illustrated in Fig. 1b, VC1 secretion was specifically induced by L-arabinose, enabling its export through the *Salmonella* fT3SS. Upon conformal attachment of the bacteria-releasing mucoadhesive sheet (BRMS) to the tumor surface and subsequent hydration, L-arabinose embedded in the central region of the BRMS activated VC1 secretion from the bacteria. Consequently, *Salmonella* released from the BRMS continuously secreted VC1 into the extracellular space via the flagellar type III secretion system (Fig. 1c). The released *Salmonella* further leveraged its intrinsic flagellar motility to actively penetrate tumor tissue, where localized VC1 delivery induced cancer cell apoptosis. Whereas the passive diffusion of conventional nanoparticles or drug molecules is severely hindered by the high interstitial fluid pressure (IFP) and dense extracellular matrix (ECM) within solid tumors, the VC1-secreting *Salmonella* released from the BRMS employs self-propulsion to migrate deeply into the hypoxic tumor core [26,27]. Consequently, the BRMS represents an advanced local therapeutic strategy that enables efficient delivery of therapeutic agents to the otherwise inaccessible tumor core by overcoming the transport limitations of passive diffusion through the biological propulsion of *Salmonella*.

### 2.2 Bacterial strains, culture, and plasmid construction

In this study, we constructed an attenuated *Salmonella* strain #1237 by introducing additional deletions of the *asd* and *rcsB* genes into *Salmonella* strain *BRD509* (*aroA aroD*), a derivative of *SL1344* (Table S1) [28]. The *rcsB* gene was deleted using λ Red–mediated targeted mutagenesis with the primers rcsB-pkd13-For and rcsB-pkd13-Re, generating the *aroA aroD rcsB* mutant strain (#245) (Tables S1 and S2) [29]. The *aroA aroD rcsB* genotype of strain #245 was subsequently introduced into the *BRD509 asd*^−^ strain (#177) via P22 phage–mediated generalized transduction [30], resulting in the construction of strain #1237 (Table S1). FlgM–Vc1.1 was designed as a translational fusion of the FlgM protein and VC1 by inserting a GS linker–6×His tag–GS linker at the C-terminus of FlgM, followed by sequential fusion of the VC1 amino acid sequence (Fig. S1a and S1b). Based on this design, the flgM-His-Vc1.1 construct was synthesized and codon-optimized by Cosmogene (Seoul, South Korea) (Fig. S1c). The synthesized DNA was digested with *Nhe*I (#1241A; Takara, Shiga, Japan) and *Sac*I (#1078A; Takara) and subsequently ligated into the pBAD18 plasmid vector under the control of the L-arabinose–inducible system [31,32]. Subsequently, *Salmonella* LT2 was used as a template for polymerase chain reaction (PCR) using the flhDC-*Sac*I-For and flhDC-*Sac*I-Re primers. The amplified flhDC fragment, including the coding sequence and ribosome binding site (RBS), was digested with *Sac*I and *Sal*I restriction enzymes (#1080A; Takara) and ligated into the flgM-Vc1.1 vector, thereby inserting the flhDC sequence downstream of the flgM-Vc1.1 sequence (Table S2). For insertion of the asd gene, the flhDC-containing plasmid was digested with the *Cla*I restriction enzyme (#1034A; Takara). The asd gene was amplified from *Salmonella* LT2 using the asd-*Cla*I-For and asd-*Cla*I-Re primers (Table S2), digested with *Cla*I, and ligated into the *Cla*I-linearized plasmid. This process resulted in the construction of the recombinant flgM-Vc1.1-flhDC-pBAD18-asd^+^ plasmid. All DNA manipulations were performed using the *Escherichia coli* strain DH5α. The completed recombinant plasmid was introduced into *S. typhimurium* SF586 and subsequently transformed into the attenuated *Salmonella* Typhimurium strain #1237 (aroA aroD rcsB asd−), generating the VC1-secreting *Salmonella* strain #1423 (Table S1).

### 2.3 Morphological and chemical characterization

The unrolled and rolled configurations of the fabricated BRMS were imaged using a digital single-lens reflex (DSLR) camera (EOS-800D, Canon). To characterize the multilayered architecture and porous microstructure of the therapeutic layer, cross-sectioned BRMS specimens were prepared and examined using a field-emission scanning electron microscope (FE-SEM, SU8020, Hitachi) at the Institute of Next-generation Semiconductor Convergence Technology (INST) of the Daegu Gyeongbuk Institute of Science and Technology (DGIST). Furthermore, the chemical compositions of the BRMS and its individual constituent layers (unrolling, guard, and therapeutic layers) were characterized using an attenuated total reflectance Fourier-transform infrared (ATR-FTIR) microscope (Nicolet Continuum, Thermo Scientific) at the INST of DGIST.

### 2.4 Unrolling test of BRMS

To evaluate the unrolling behavior of the BRMS in a moist physiological environment, an *ex vivo* unrolling test was conducted using porcine small intestinal tissue (BIOZOA Biological Supply). The rolled BRMS was placed onto the tissue surface, and the unrolling process, driven by the swelling of the unrolling layer, was continuously recorded using a DSLR camera. Based on the recorded video footage, the time-resolved evolution of the BRMS unrolling behavior was systematically analyzed.

### 2.5 *In vitro* VC1 releasing profile from BRMS

An *in vitro* release study was performed to characterize the time-dependent secretion profile of the anticancer protein VC1 produced by the VC1-secreting *Salmonella* encapsulated within the BRMS. Luria-Bertani (LB) broth was used as the release medium to maintain bacterial viability and metabolic activity, thereby supporting continuous protein secretion. The BRMS was immersed in LB broth and incubated at 37°C. The experimental groups were divided into two conditions: one with an additional external supply of L-arabinose and the other without external L-arabinose, relying solely on the arabinose pre-encapsulated within the BRMS to induce VC1 expression. At predetermined time intervals (0, 4, 8, 12, and 24 h), culture supernatants were collected and quantitatively analyzed using a His-tag ELISA Detection Kit (#L00436, GenScript, NJ, USA), which specifically detects the His-tag fused to the VC1 protein. Absorbance value was measured using a UV-visible spectrophotometer (SPECTROstar Nano, BMG Labtech), and the cumulative VC1 release over time was calculated by interpolating the measured values against a standard calibration curve.

### 2.6 Tissue adhesion force of BRMS

The mucoadhesive force between the fabricated BRMS and porcine small intestinal tissue (BIOZOA Biological Supply, Korea) was quantitatively measured using a motorized linear stage (KZL06050-N1-FD, Suruga Seiki, Japan) and a load cell (DBCM-5, Bongshin, Korea). All experiments were conducted under the assumption that the BRMS was fully unrolled. First, the load cell was fixed to the upper mount, and the porcine small intestine was securely fixed onto the motorized stage. Subsequently, the fully unrolled BRMS was connected to the load cell via a string and gently placed on the surface of the porcine small intestine. After maintaining contact for a predefined duration, the stage was lowered at a constant speed to measure the detachment force transmitted to the load cell. The force data were continuously recorded using a LabVIEW-based acquisition system (National Instruments, USA). A gelatin sheet, commonly used in drug delivery studies, was selected as a control for comparative evaluation of adhesion forces. The gelatin sheet was fabricated by adapting a previously reported method [33]. Briefly, a 10 wt% gelatin (from bovine skin, Duksan, Korea) solution was cast into a 35 mm dish and dried overnight; the resulting gelatin sheet was then cut to the same dimensions as the BRMS (7 mm × 10 mm). Both the BRMS and the gelatin control sheet were allowed to contact the porcine small intestinal surface for 60 s and 120 s, respectively, after which the detachment forces were measured by lowering the motorized stage.

### 2.7 *In vitro* mucus penetration and retention assay

To evaluate the mucosal adhesion and active bacterial penetration from the BRMS under conditions that mimic GI fluid flow, a Transwell-based assay simulating the GI mucosal interface was performed. The transmembrane insert of the upper chamber in a 12-well Transwell system (pore size: 3.0 μm, Corning, USA) was uniformly coated with 0.5% motility agar to reproduce the physical barrier functions and viscoelastic properties of the mucus layer covering the GI epithelium and tumor surface [34]. For the experimental group, the BRMS was firmly attached to the agar surface, whereas the control group was inoculated with a free bacterial suspension containing an equivalent bacterial load (10^7^ CFU). To simulate the wash-out effect caused by intestinal peristalsis and luminal fluid flow, a physical washing step was introduced. After a 1-min incubation period, the medium in the upper chamber was removed by pipetting to eliminate any unattached BRMS or bacteria and was subsequently replaced with fresh PBS. During incubation at 37°C, aliquots of the medium from the lower chamber were collected at predetermined time intervals. The number of bacteria penetrating the mucus-mimicking layer was quantified by enumerating colony-forming units (CFU) using Petrifilm plates (Neogen, USA).

### 2.8 Formation of CT-26 tumor spheroids

To replicate the dense three-dimensional (3D) architecture and microenvironment of native solid tumors, tumor spheroids were generated using CT-26 mouse colon carcinoma cells. CT-26 cells were seeded into 96-well round-bottom ultra-low attachment plates (Corning, USA) at a density of 1 × 10^4^ cells per well. To facilitate cell aggregation, the plates were centrifuged at 1300 rpm for 5 min. Subsequently, the cells were incubated at 37°C in a humidified atmosphere containing 5% CO_2_ until the spheroids reached an average diameter of approximately 400 μm.

### 2.9 Evaluation of tumor tissue penetration of *Salmonella* in BRMS

To evaluate the intratumoral penetration efficiency of therapeutic agents, an *in vitro* tumor penetration test was performed using the preformed CT-26 tumor spheroids. The experimental group was treated with a BRMS loaded with Cy5-labeled *Salmonella* (10^7^ CFU), while the control group was treated with a sheet loaded with doxorubicin (DOX). The DOX-loaded sheet was fabricated by substituting the therapeutic layer of the BRMS with a chitosan-catechol layer containing the anticancer drug DOX. Briefly, chitosan-catechol, synthesized according to a previously reported protocol [35], was dissolved in deionized (DI) water at a concentration of 2 wt% and mixed with DOX to a final concentration of 0.1 mg/mL. The resulting solution was cast onto the TEGDM layer of a 2-layer structure (unrolling/guard layers) and lyophilized at −80°C. The freeze-dried sheet was then cut to the same dimensions as the BRMS (10 × 7 mm^2^) for experimental use. For the penetration test, the CT-26 spheroids were placed in the lower chamber of a Transwell system coated with 0.5% motility agar. Subsequently, the BRMS and the DOX-loaded sheet were placed on the motility agar and incubated for 4 h. The spheroids were then collected, washed with PBS, and fixed with 4% paraformaldehyde (PFA) for 30 min. Cell nuclei were stained with DAPI, and the penetration depth and spatial distribution of DOX and bacteria (Cy5 signal) within the CT-26 tumor spheroids were analyzed by acquiring Z-stack images from the spheroid surface to the core using a confocal laser scanning microscope (LSM900, Carl Zeiss) at the INST of DGIST. In addition, the intratumoral penetration performance of DOX and bacteria in the spheroid was quantified using a custom image processing algorithm, developed in Python (SI Section 1).

### 2.10 *In vitro* cancer cell killing evaluation under physiological-mimetic conditions

To evaluate the cancer cell-killing efficacy of the BRMS under conditions recapitulating physiological physical barriers and fluid flow, an *in vitro* model was established using a 12-well Transwell system (pore size: 3.0 μm, Corning, USA). CT-26 colon cancer cells were seeded into the lower chamber at a density of 10^5^ cells/well and incubated at 37°C with 5% CO_2_ for 24 h to allow for stable attachment. To mimic the physical resistance of the GI mucus and tumor tissue, the upper chamber was coated with 0.5% motility agar. The experimental groups were categorized as follows: (1) control, (2) DOX-loaded sheet, (3) free *Salmonella* suspension (10^7^ CFU/mL), (4) carrier-only control [BRMS (-Sal)], (5) BRMS loaded with non-induced *Salmonella* [BRMS (+Sal, -VC1)], and (6) the fully functional VC1-secreting BRMS [BRMS (+Sal, +VC1)]. To simulate the loss of therapeutic agents and bacteria caused by peristalsis and GI fluid flow, each sample was applied onto the motility agar layer in the upper chamber. After 1 min of application, a wash-out step was performed by removing the medium and washing out the chamber. Subsequently, fresh medium was replenished, and the cells were incubated for an additional 24 h. Following incubation, the viability of the cancer cells in the lower chamber was assessed using a Live/Dead viability kit (Invitrogen, USA). The stained cells were visualized using a confocal laser scanning microscope (LSM900, Carl Zeiss, Germany). Therapeutic efficacy was qualitatively and quantitatively evaluated by analyzing the fluorescence signals of live (green, Calcein-AM) and dead (red, EthD-1) cells.

### 2.11 *Ex vivo* BRMS delivery test using a colonoscope

To evaluate the delivery and deployment performance of the BRMS using a clinically relevant standard colonoscope, an *ex vivo* experiment was performed. First, fresh porcine small intestinal tissue (BIOZOA Biological Supply, South Korea) was mounted inside a custom 3D-printed intestine phantom designed to replicate the curvature and morphology of the GI tract. A commercial colonoscope (CF-V70L, Olympus) was used for the experiment. To protect the BRMS from damage or premature loss caused by the influx of intestinal fluids during endoscopic navigation, a custom-fabricated Ecoflex cap was attached to the distal tip of the endoscope. The design specifications and fabrication process of the Ecoflex cap are provided in Fig. S2. In the locomotion mode, the elastic restoring force of the elastomer maintains the Ecoflex cap in a closed configuration, thereby physically blocking fluid ingress into the working channel during insertion and preserving the BRMS in a dry, rolled state. Conversely, in the BRMS delivery mode, which is initiated upon reaching the target site, the mechanical force exerted by the forceps pushing the BRMS causes the Ecoflex cap to temporarily deform and open, allowing the BRMS to be released from the working channel. This Ecoflex cap was designed to maintain a closed state under normal conditions to protect the BRMS inside the working channel and to open only upon the application of mechanical force during BRMS deployment. The BRMS was loaded into the working channel of the endoscope in a rolled state, followed by biopsy forceps positioned proximally to push the BRMS. After advancing the endoscope through the phantom to reach the target lesion site, the forceps were advanced through the working channel to apply controlled mechanical force. This force caused the Ecoflex cap to elastically expand and open, thereby deploying the BRMS onto the target lesion site; immediately after deployment, the BRMS absorbed fluids from the tissue, self-unrolled, and adhered conformally to the tissue surface. These processes were recorded in real-time using the colonoscopic imaging system. To further validate delivery feasibility under conditions more closely resembling the clinical setting, an additional *ex vivo* intraluminal experiment was conducted in which the endoscope was inserted into the intact lumen of a porcine small intestine without surgical incision. In this experiment, following endoscope insertion into the lumen, a single BRMS was successfully deployed onto the inner intestinal wall using the same Ecoflex cap and forceps-based delivery mechanism. The intraluminal release, self-unrolling, and adhesion behavior of the BRMS were recorded within the endoscopic field of view, confirming robust and precise deployment under realistic GI lumen conditions.

### 2.12 *Ex vivo* BRMS delivery test using a magnetically actuated capsule robot

We conducted an *ex vivo* experiment to demonstrate BRMS delivery using a custom magnetically actuated capsule robot developed in our laboratory. The experimental environment was designed to mimic physiological intestinal conditions by mounting porcine small intestinal tissue within a custom 3D-printed phantom. The capsule robot employed in this experiment was fabricated based on a previously reported design [36,37], with structural modifications specifically implemented to meet the requirements of the present work. The primary components of the capsule, schematically illustrated in Fig. S3a, include a capsule body, a center rod, a driving unit incorporating a permanent magnet and a pushing bar, and a capsule head. Notably, a flexible Ecoflex lids assembly was integrated at the anterior end of the capsule; this component was designed to block the ingress of intestinal fluids during locomotion, thereby protecting the loaded BRMS. The detailed geometry and fabrication procedure of the Ecoflex lids assembly are provided in Fig. S4. In locomotion mode, the anterior Ecoflex lids assembly remains closed, thereby sealing and protecting the loaded BRMS from external intestinal fluids. In contrast, in the delivery mode, an internal mechanical actuation mechanism opens the lids assembly, enabling controlled release of the BRMS. Precise wireless control of the capsule robot was achieved using an electromagnetic actuation (EMA) system composed of six coils. This system generates uniform and gradient magnetic fields in three-dimensional space, enabling accurate control of the capsule position and orientation; the underlying magnetic actuation principles were described in our previous study [36]. For the experiment, four BRMS units in a rolled configuration were loaded into the storage compartment of the capsule robot. Initially, in locomotion mode, the capsule was navigated to the target location under EMA control, after which it was switched to BRMS delivery mode to deploy the BRMS at the target site. During deployment, the internal driving unit rotated about the center rod and translated forward, thereby mechanically opening the Ecoflex lid and delivering the BRMS (Fig. S3). This procedure was repeated sequentially to deliver four BRMS units to spatially distinct target locations, and the entire delivery process was recorded using an external DSLR camera.

### 2.13 *In vivo* antitumor efficacy evaluation

All animal procedures were performed in accordance with the guidelines approved by the KIST Institutional Animal Care and Use Committee (IACUC) (Approval No.: 2025-032). Five-week-old female BALB/c mice were purchased from OrientBio (Seongnam, Korea) and acclimatized for one week. For the establishment of a subcutaneous tumor model, 1 × 10^6^ cells of CT-26 murine colon carcinoma cells in 30 μL per mouse were inoculated into the left flank on Day −14. Tumor size was measured daily, and treatment was initiated when tumor volumes reached approximately 85-130 mm^3^ (Day 0). Mice were randomized into five groups (n = 5 per group): G1 (PBS), receiving PBS as a negative control; G2 (Free *Salmonella* suspension), in which an equivalent dose of VC1-secreting *Salmonella* suspension was applied directly onto the tumor surface without the BRMS; G3 (BRMS (−Sal)), implanted with BRMS without VC1-secreting *Salmonella*; G4 (BRMS (+Sal, −VC1)), implanted with BRMS loaded with VC1-secreting *Salmonella* without induction of VC1 expression; and G5 (BRMS (+Sal, +VC1)), implanted with BRMS loaded with VC1-secreting *Salmonella* and subjected to arabinose induction to activate VC1 secretion.

On Day 0, mice were anesthetized using TerrellTM isoflurane (Piramal, PA, USA), and a small incision was made adjacent to the tumor. For G1, PBS was administered locally at the corresponding site; for G2, an equivalent dose of the VC1-secreting *Salmonella* suspension was applied directly onto the exposed tumor surface without the sheet; and for G3–G5, a BRMS sheet was positioned to cover the tumor surface and secured by suturing. For induction of VC1 secretion in G5, 200 μL of 40% (w/v) L-arabinose was intraperitoneally injected once daily from Day 0 to Day 12. Body weight and tumor dimensions were recorded daily throughout the treatment period. Tumor volume was calculated using the formula: V = 0.5×length×width^2^. On Day 14, mice were euthanized, and tumors were harvested for histological analysis and weight measurement. For histological evaluation, excised tumors from each group were paraffin-embedded (39601006, Leica Biosystems), and sectioned at 4 μm thickness. For morphological evaluation, sections were stained with hematoxylin (20032, Muto Pure Chemicals) and eosin (H&E; 17025S1210, Junsei Chemical). Apoptosis was assessed using terminal deoxynucleotidyl transferase dUTP nick-end labeling (TUNEL) with a TUNEL Assay Kit–HRP–DAB (AB206386, Abcam). Immunoreactivity was visualized using a diaminobenzidine (DAB) detection system (K3468, Dako).

### 2.14 Statistical analysis

All statistical data analyses were performed with Microsoft Excel. Data are expressed as mean ± standard deviation (SD) from at least three independent experiments. Statistical significance among groups was analyzed through the t-test and one-way analysis of variance (ANOVA). P-values less than 0.005 were considered statistically significant (∗: <0.005, ∗∗: <0.0005, ∗∗: <0.005, ∗∗∗: <0.0001, and ns: ≥ 0.05).

## 3 Results and discussion

### 3.1 Characterization of BRMS

The designed BRMS was fabricated using a standard molding process followed by UV-induced photopolymerization, as detailed in the Supporting Information (SI Section 2, Fig. S5). Images of the fabricated BRMS in its fully unrolled and completely rolled states are presented in Fig. 2a and b, respectively. The unrolled BRMS measured approximately 10 mm × 7 mm (width × length) and was subsequently rolled for loading into endoscopic devices and capsule robots. Fig. 2c presents a cross-sectional SEM image of the BRMS. The SEM analysis verified the formation of a well-defined multilayered architecture, with the unrolling layer, guard layer, and therapeutic layer (central and peripheral regions) clearly distinguishable. The thicknesses of these layers were measured to be approximately 16 μm, 8 μm, and 0.7 mm, respectively. In particular, a highly porous microstructure, advantageous for enhanced mucoadhesion, was clearly observed in the magnified view of the therapeutic layer.

**Fig. 2:**
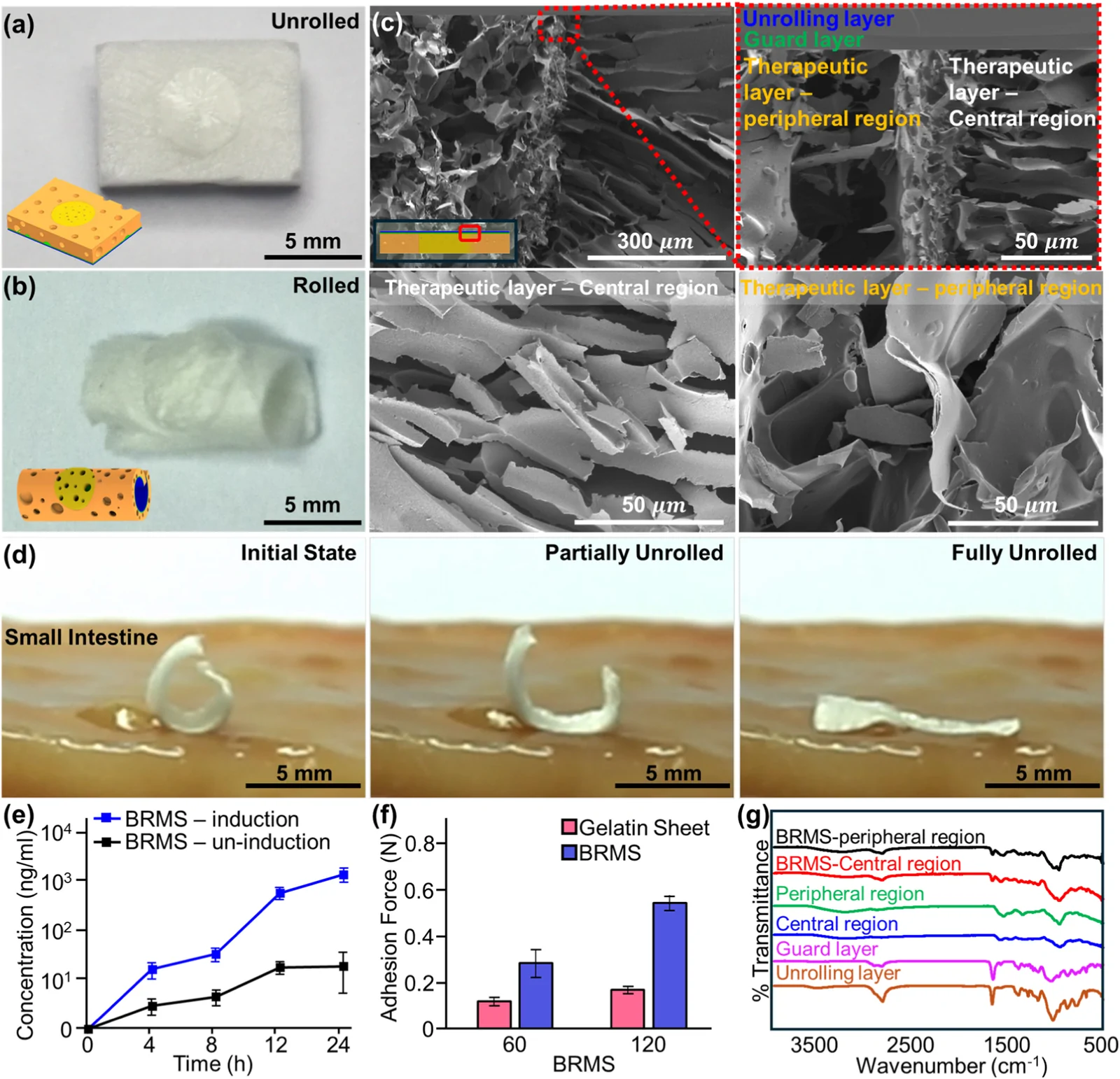
Characterization of the multi-layered BRMS. (a, b) Representative optical images illustrating the BRMS in its (a) fully unrolled and (b) compactly rolled states. (c) Cross-sectional SEM images revealing a well-defined three-layer architecture consisting of the unrolling, guard, and therapeutic layers, each exhibiting a porous microstructure. (d) Time-lapse images demonstrating the hydration-triggered self-unrolling behavior of the BRMS when deployed on *ex vivo* porcine small intestinal tissue. (e) Time-dependent VC1 release profiles from the BRMS under L-arabinose-induced conditions (n = 5). (f) Quantitative comparison of the mucoadhesive strength of the BRMS versus a gelatin control sheet (n = 10). (g) FTIR spectra of the individual unrolling, guard, and therapeutic layers, as well as the assembled BRMS, confirming material composition and successful multilayer integration.

The unrolling process of the BRMS on the surface of *ex vivo* small intestinal tissue is presented in Fig. 2d. When the rolled BRMS is placed on the small intestinal mucosa pre-wetted with intestinal fluids, the unrolling layer rapidly absorbs the fluid and begins to swell anisotropically. The resulting swelling-induced bending moment and stress relaxation collectively drive the unrolling of the BRMS [38]. The BRMS progressively unrolled from its initial state, becoming fully unrolled within 100 s and forming conformal contact with the small intestinal surface (Movie S1).

Fig. 2e presents the evaluation of the VC1 secretion capability of the *Salmonella* encapsulated within the BRMS. The experiments were conducted on LB broth media, comparing secretion levels with and without an additional external supply of L-arabinose. Quantitative His-tag ELISA analysis revealed that the group receiving supplemental L-arabinose in the medium exhibited a high mean VC1 concentration of 1356.5 ng/mL after 24 h. In contrast, the group relying exclusively on the L-arabinose pre-encapsulated within the central region of the BRMS, without external supplementation, showed a substantially lower average VC1 concentration of 18.7 ng/mL after 24 h. These results indicate that the amount of L-arabinose loadable within the current BRMS dimensions was insufficient to induce sustained, high-level protein expression. Although the current BRMS design demonstrated limitations in achieving autonomous high-concentration VC1 release due to restricted inducer loading capacity, this challenge can be readily addressed in future iterations. Potential strategies include engineering *Salmonella* strains capable of constitutive VC1 secretion without external stimulation or employing bacteria with tumor microenvironment-responsive promoters (e.g., hypoxia- or acidic pH-responsive), thereby enabling sustained, high-efficiency VC1 release without a separate chemical inducer [39,40].

Fig. 2f presents a comparative evaluation of mucoadhesive force between the control gelatin sheet and the BRMS, measured using a load cell coupled with a motor stage. After an attachment time of approximately 60 s on the small intestinal tissue surface, the adhesion forces of the gelatin sheet and the BRMS were measured to be 0.118 N and 0.283 N, respectively. When the attachment time was extended to 120 s, the adhesion force of the gelatin sheet increased slightly to 0.135 N, whereas that of the BRMS increased substantially to 0.544 N, demonstrating an adhesion strength approximately 4.03-fold greater than that of the control. These results indicate that the mucoadhesive components within the peripheral region of the therapeutic layer form strong physicochemical interactions with the tissue surface, thereby providing robust adhesion capable of withstanding intestinal peristalsis and continuous luminal fluid flow.

Finally, the chemical composition of the BRMS and its individual layers was characterized by Fourier transform infrared (FTIR) spectroscopy (Fig. 2g). The spectra obtained from the peripheral and central regions of the BRMS exhibited all major characteristic absorption peaks corresponding to the unrolling, guard, and therapeutic layers. These results confirm that no chemical denaturation or degradation occurred during the multilayer stacking and fabrication processes, thereby chemically validating that each layer maintains its structural integrity to independently fulfill its intrinsic function. A comprehensive peak analysis of the FTIR spectra is provided in the Supporting Information (SI Section 3).

### 3.2 Bacterial tumor penetration in BRMS

To validate the active deep intratumoral penetration capability of the bacteria, which underpins the core therapeutic mechanism of the BRMS, we employed a Transwell-based model mimicking the GI mucus barrier and a three-dimensional (3D) multicellular tumor spheroid model that recapitulates the architecture of native tumor tissue.

The bacterial penetration and retention under mucus-mimicking conditions were evaluated using a Transwell-based model (Fig. 3a). Following the wash-out step, the control bacterial suspension exhibited rapid loss, resulting in a negligible bacterial count (∼2.76 × 10^4^ CFU/mL) in the lower chamber after 24 h. In contrast, the BRMS maintained stable adhesion and enabled significantly enhanced bacterial penetration (∼3.60 × 10^6^ CFU/mL), indicating markedly improved retention and transport efficiency. This enhanced performance is attributed to the strong mucoadhesion of the peripheral region, which anchors the BRMS to the surface under fluidic conditions, while the guard layer prevents bacterial loss toward the lumen, enabling localized and directional release.

**Fig. 3:**
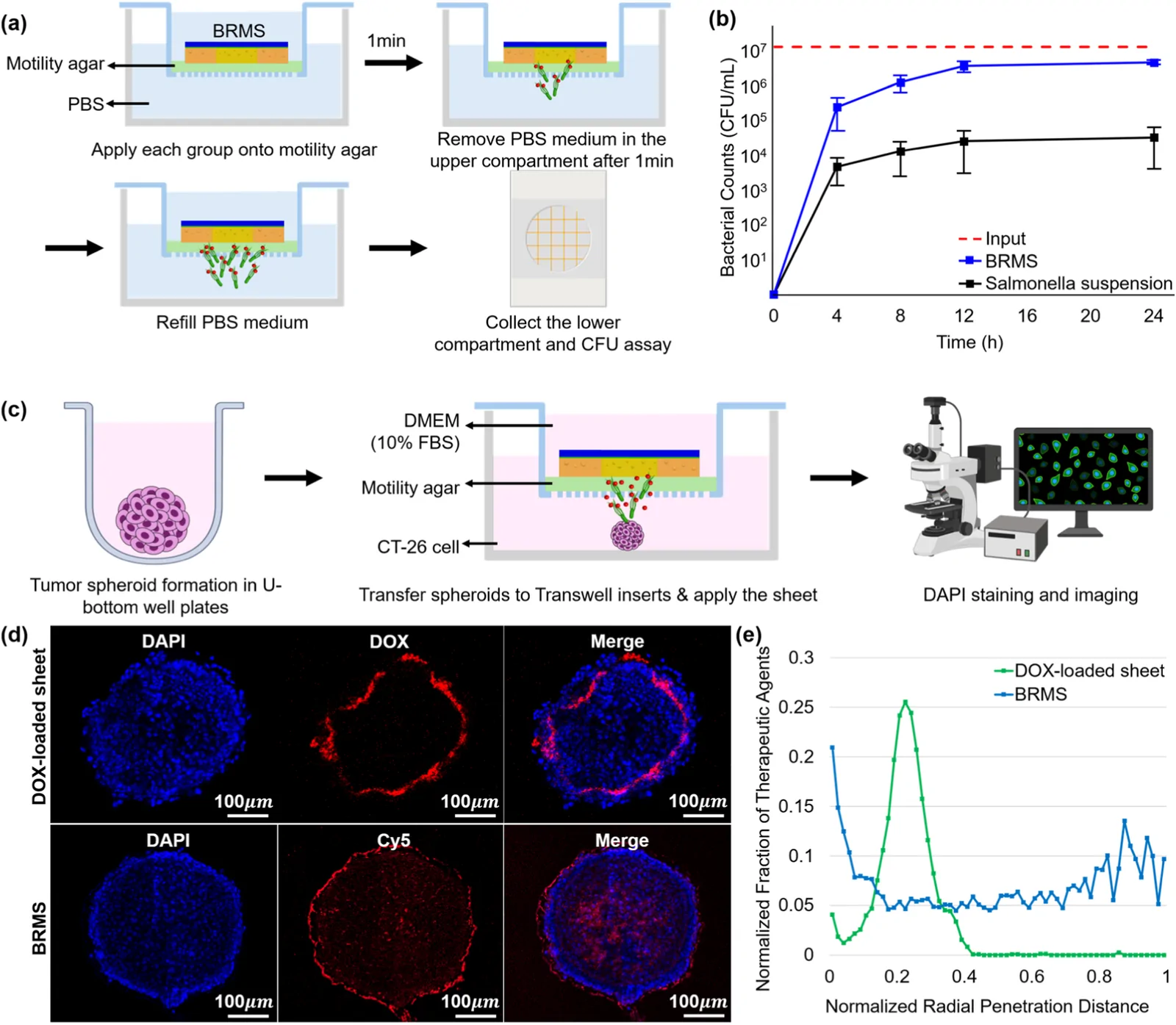
Evaluation of the bacterial penetration efficiency and deep intratumoral infiltration mediated by the BRMS. (a) Schematic illustration of the *in vitro* transmembrane penetration assay using a Transwell system integrated with a motility agar layer to recapitulate biological and physical transport barriers. (b) Quantitative assessment of bacterial penetration into the lower compartment over a 24 h period, comparing BRMS-mediated delivery with a free *Salmonella* suspension (n = 5). (c) Schematic representation of the 3D tumor spheroid infiltration assay, in which CT-26 spheroids were positioned beneath a motility agar layer to assess bacterial penetration across a dense physical barrier. (d) Representative confocal microscopy images of CT-26 tumor spheroids treated with a DOX-loaded sheet and the BRMS. Blue fluorescence denotes cell nuclei (DAPI), while red fluorescence corresponds to doxorubicin (DOX) in the top row and Cy5-labeled *Salmonella* in the bottom row, highlighting the peripheral confinement of the drug control and the markedly enhanced deep intratumoral infiltration and accumulation achieved by the BRMS. (e) Radial signal-positive area profile of (d), plotted against normalized radial penetration distance (0 = surface, 1 = centroid). The DOX-loaded sheet remains confined to the periphery, whereas the BRMS shows a signal-positive area extending into the spheroid core, indicating deep intratumoral infiltration.

To further investigate intratumoral penetration, a 3D CT-26 tumor spheroid model was employed. Confocal microscopy analysis revealed that DOX delivery was restricted to the outer region of the spheroid, indicating limited penetration due to diffusion constraints. In contrast, *Salmonella* released from the BRMS actively penetrated the spheroid and distributed throughout the tumor core, demonstrating significantly enhanced intratumoral transport. This behavior is attributed to the flagella-driven motility and tumor-targeting characteristics of *Salmonella*. Subsequently, driven by their intrinsic anaerobic preference and chemotactic behavior, the bacteria deeply infiltrated the hypoxic tumor core and were distributed more uniformly throughout the tumor tissue [12]. To quantitatively substantiate this observation, we analyzed the signal-positive area profile of the confocal images of the spheroids as a function of the normalized radial penetration distance (Fig. 3e). As shown in Fig. 3e, the DOX-loaded sheet exhibited a single peak at approximately 21% depth from the spheroid surface before attenuating to nearly zero toward the center, whereas the BRMS showed signal-positive area distributed across the entire radius. This demonstrates that, owing to the active motility of *Salmonella*, the BRMS can overcome the limitations of passive diffusion and enable bacterial penetration into the tumor core. Overall, the BRMS enables both stable localization under GI fluid conditions and active deep tissue penetration, overcoming the limitations of passive diffusion-based delivery systems.

### 3.3 *In vitro* cytotoxicity evaluation of the BRMS

Before evaluating the therapeutic efficacy of the bacteria-releasing mucoadhesive sheet (BRMS), we assessed whether the genetically engineered *Salmonella* loaded within the BRMS could efficiently express and secrete the therapeutic protein VC1. Fig. 4a shows the plasmid map designed for L-arabinose–inducible expression of VC1. The engineered attenuated *Salmonella* was designed to express VC1 under the control of the L-arabinose-inducible AraC/pBAD system, which enables regulated expression of the downstream flgM–VC1 fusion gene (Fig. 4a; Fig. S1) [31,41]. The resulting flgM–VC1 fusion protein is subsequently exported through the flagellar type III secretion system (fT3SS), exploiting the intrinsic export property of flgM as a native substrate of the flagellar secretion apparatus [42,43]. This flgM-based fT3SS strategy has also been previously applied by our group for secretion of other conotoxin peptides with anticancer activity [44,45]. As shown in the Western blot analysis in Fig. 4b, VC1 protein was clearly detected in both bacterial cell lysates and culture supernatants after 6 h of induction with L-arabinose. Importantly, the cytosolic marker protein DnaK was not detected in the culture supernatant (Fig. 4b), indicating that VC1 release was not due to bacterial lysis but rather occurred through an active secretion mechanism. To further quantify the amount of secreted VC1, culture supernatants collected after 6 h of L-arabinose induction were analyzed by His-tag ELISA. Only a low basal level of VC1 was detected under non-induced conditions (∼10.5 ng/mL), whereas L-arabinose induction markedly increased secreted VC1 to ∼181.9 ng/mL, corresponding to an approximately 17.3-fold increase (Fig. S6). Collectively, these results demonstrate that the engineered *Salmonella*–based fT3SS enables efficient and controllable extracellular secretion of the therapeutic protein VC1, highlighting its robustness and suitability as a bacterial protein secretion platform. To verify the biological activity of secreted VC1, CT-26 cells were exposed to test samples under various experimental conditions, and cytotoxicity was systematically evaluated (Fig. 4c). Minimal cytotoxicity was observed in the PBS control and in cells treated with culture supernatant from *Salmonella* under non-inducing conditions (−VC1). In contrast, culture supernatant collected under VC1-inducing conditions (+VC1) caused partial loss of cell viability, indicating that VC1 secreted from *Salmonella* retains its anticancer activity. When live bacteria were applied directly, the *Salmonella* suspension without VC1 induction (+Sal, −VC1) produced moderate cell death attributable to intrinsic bacterial cytotoxicity; however, the VC1-induced *Salmonella* suspension (+Sal, +VC1) elicited a markedly enhanced cytotoxic effect and yielded the highest cancer cell death rate. To determine whether the cytotoxic activity of secreted VC1 extends to human colorectal cancer, additional viability analyses were performed using multiple colon cancer cell lines (CT-26, SW480, HT-29) treated with *Salmonella* culture supernatants (Fig. S7). Bright-field microscopy at 48 h post-treatment showed that the VC1-induced supernatant (+VC1) substantially reduced cell density and induced pronounced morphological damage consistent with cell death, compared with PBS and non-induced supernatant (−VC1) controls (Fig. S7a). Quantitative WST-8 assay at 24 h further demonstrated significantly decreased cell viability in the +VC1-treated group relative to controls across all tested lines, including both mouse-derived CT-26 and human-derived SW480 and HT-29 cells (Fig. S7b). Collectively, these findings indicate that the engineered *Salmonella*-based VC1 secretion platform mediates broad, potent, and cross-species cytotoxicity against colorectal cancer cells.

**Fig. 4:**
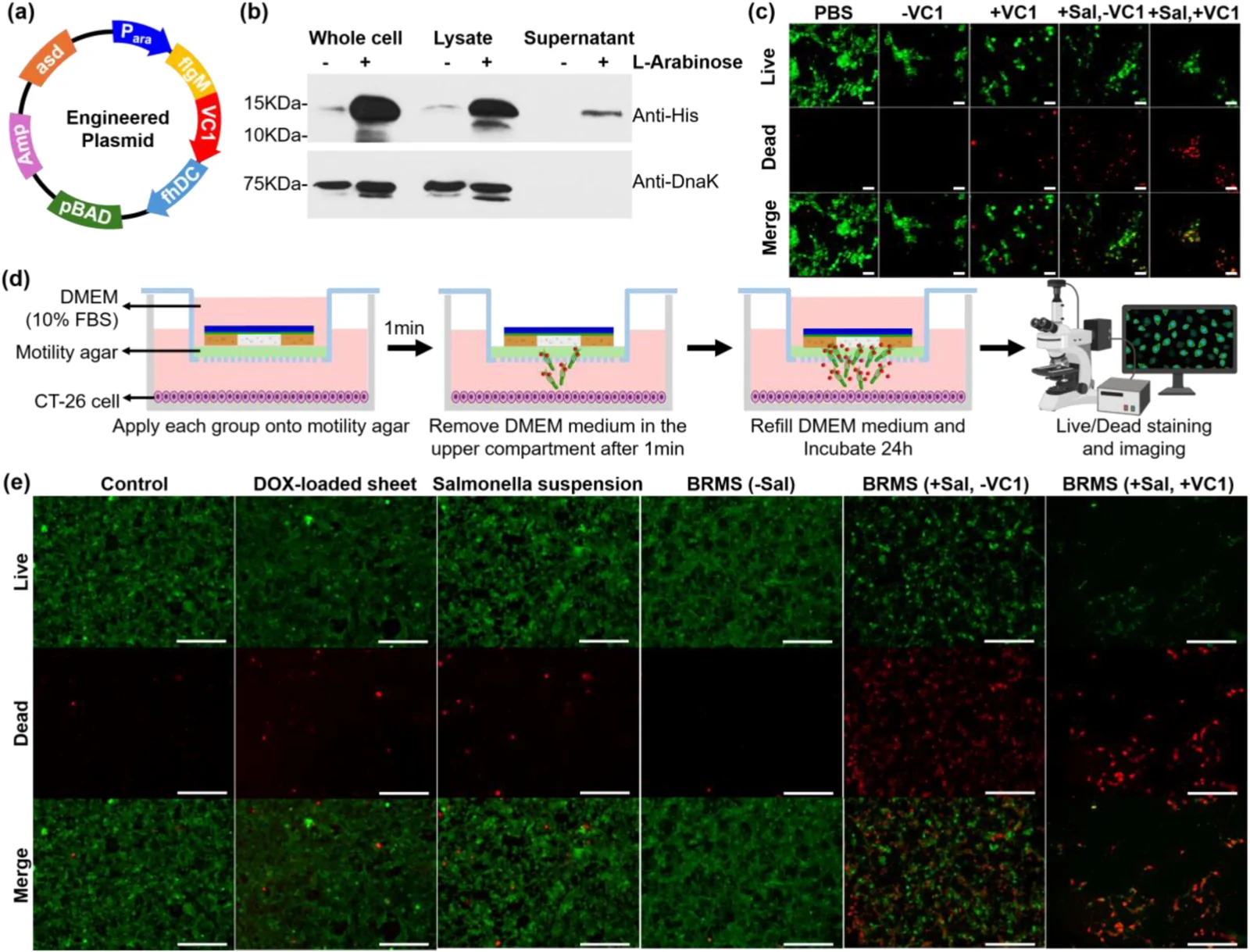
Construction and functional validation of the engineered *Salmonella*-based VC1 secretion system and *in vitro* evaluation of BRMS therapeutic efficacy. (a) Schematic representation of the plasmid architecture (flgM–VC1–flhDC–pBAD18–asd), which enables L-arabinose–inducible VC1 expression and active extracellular secretion via the flagellar type III secretion system. (b) Western blot analysis confirming VC1 expression and secretion. VC1-secreting *Salmonella* were induced with L-arabinose for 6 h, and VC1 and DnaK proteins were detected in whole cell fractions, cell lysates, and culture supernatants using anti-His and anti-DnaK antibodies, respectively. (c) Live/Dead cytotoxicity assay of CT-26 cells treated with conditioned media from different bacterial groups to verify the biological activity of secreted VC1. Scale bars are 20 μm. (d) Schematic illustration of the *in vitro* therapeutic efficacy assay using a Transwell system, in which CT-26 cells were physically separated from the treatment groups by a motility agar layer to recapitulate physical barriers and wash-out conditions. (e) Representative fluorescence microscopy images of CT-26 cells stained with Live (green)/Dead (red) kit after 24 h of treatment with different groups: untreated control, DOX-loaded sheet, free *Salmonella* suspension, and three BRMS formulations, including a carrier-only control [BRMS (−Sal)], a bacteria-loaded carrier without VC1 induction [BRMS (+Sal, −VC1)], and the fully functional VC1-secreting BRMS [BRMS (+Sal, +VC1)]. Scale bars are 500 μm.

Based on this validation, we performed a cytotoxicity assessment using CT-26 cells to evaluate cancer cell-killing efficacy under conditions that recapitulate physiological barriers and fluid flow (Fig. 4d). As shown in Fig. 4e, the control group exhibited high cell viability, with most cells displaying green fluorescence (live). The DOX-loaded sheet group showed minimal cytotoxicity, despite containing an anticancer drug. This outcome is attributed to the dependence of DOX on passive diffusion, which prevented effective penetration across the upper motility agar barrier and limited drug accumulation in the cancer cell layer within the lower chamber. The free *Salmonella* suspension group also showed high cancer cell survival, demonstrating that the suspension-based bacterial delivery is ineffective in environments where physical barriers and fluid flow coexist, as most bacteria are removed during the wash-out process and fail to penetrate the barrier. The BRMS without *Salmonella* (BRMS (-Sal)) group exhibited cell viability comparable to the control, confirming that the polymeric components of the empty BRMS are highly biocompatible and do not induce cytotoxicity [33,46,47]. The BRMS loaded with non-induced *Salmonella* (BRMS (+Sal, -VC1)) group showed moderate cytotoxicity, with a substantial fraction of cells displaying red fluorescence (dead), even in the absence of VC1 induction. This suggests that *Salmonella* released from the BRMS, which remained attached during wash-out, successfully penetrated the agar barrier and suppressed cancer cell viability through intrinsic oncolytic activity and nutrient competition associated with bacterial proliferation [48,49]. The BRMS loaded with VC1-induced *Salmonella* (BRMS (+Sal, +VC1)) group demonstrated the most pronounced and robust cancer cell-killing effect among all groups. This enhanced efficacy is attributed to the synergistic action of *Salmonella's* intrinsic cytotoxicity and the anticancer activity of VC1; bacteria released from the strongly mucoadhesive BRMS actively penetrated the barrier and secreted VC1 in proximity to the cancer cells. In conclusion, these results demonstrate that the VC1-induced *Salmonella*-loaded BRMS functions as a highly effective therapeutic platform, capable of targeting and eliminating cancer cells even under challenging conditions involving physical barriers and continuous fluid flow.

### 3.4 *Ex vivo* BRMS delivery

To validate the safety, precision, and reliability of BRMS delivery to target lesions within the GI tract, we performed *ex vivo* delivery experiments using porcine small intestinal tissue, which closely resembles the anatomy of the human GI tract. First, we validated the BRMS delivery strategy using a colonoscope, a clinically established medical device widely employed in routine practice. A rolled BRMS was loaded within the working channel of the endoscope and delivered to the porcine small intestinal surface. To address fluid-induced damage or premature loss, a soft Ecoflex cap was attached to the distal tip of the endoscope (Fig. S2). Fig. 5a and b illustrate the locomotion mode and the forceps-assisted delivery mode of the colonoscope equipped with the BRMS-loaded working channel and the Ecoflex cap. Figure S2a and Fig. 5c show the endoscopic system configuration and the control handle with the forceps inserted into the working channel, respectively. The experimental setup was established by fixing porcine small intestinal tissue within a custom 3D-printed phantom, as shown in Fig. 5d. Upon reaching the target location, the forceps were carefully manipulated to release the BRMS; the four deployed BRMS units rapidly absorbed fluids from the tissue surface, immediately self-unrolled, and firmly adhered to the small intestinal mucosa (Fig. 5e). The release process of the BRMS from the working channel and its subsequent unrolling behavior are shown in Fig. S8 and Movie S2, respectively. Additionally, to closely mimic the native GI lumen environment, we performed an experiment in which the endoscope was inserted into the intact lumen of a porcine small intestine without surgical incision to deliver the BRMS (Fig. S8 and Movie S3). In this experiment, the successful deployment of a single BRMS further confirmed the feasibility of endoscopic BRMS delivery within the actual intestinal lumen.

**Fig. 5:**
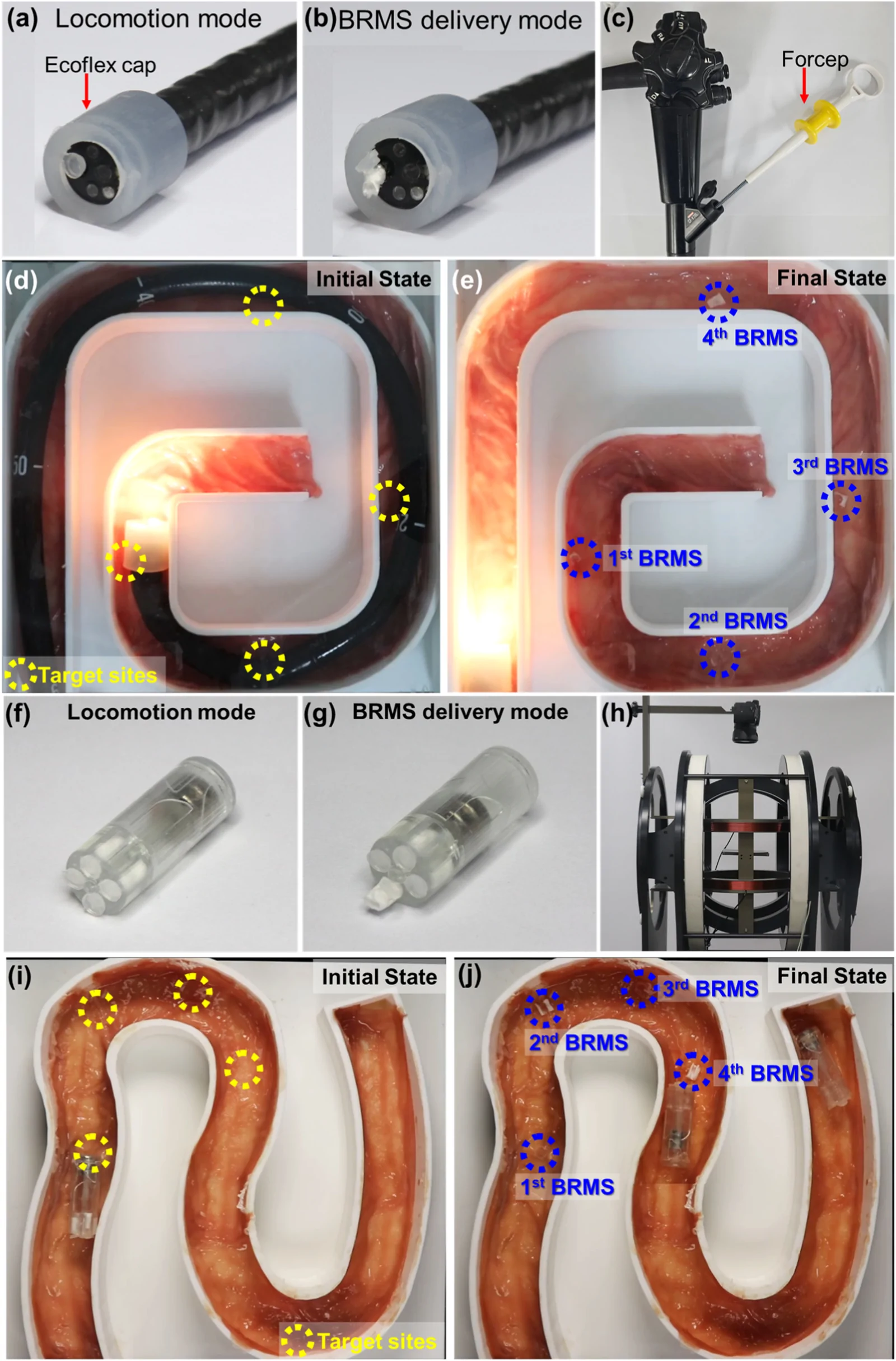
*Ex vivo* validation of BRMS delivery using a clinical endoscope and a magnetically actuated capsule robot. Schematic representations of the endoscope-assisted delivery system in (a) locomotion mode and (b) BRMS delivery mode. The distal Ecoflex cap remains sealed to prevent fluid ingress during navigation and elastically deforms to release the BRMS upon mechanical pushing by endoscopic forceps. (c) Photographic images illustrating the endoscopic control handle and the insertion of forceps through the working channel. (d) Experimental setup employing a porcine small intestine phantom model. (e) Successful endoscopic release, self-unrolling, and conformal adhesion of the BRMS on the intestinal mucosal surface. (f, g) Schematic illustrations of the magnetically actuated capsule robot operating in (f) locomotion mode and (g) BRMS delivery mode. (h) Photograph of the EMA system consisting of 6 coils. (i) Experimental setup for capsule-mediated BRMS delivery in the porcine intestine phantom. (j) Demonstration of multi-site delivery, showing the final state after four individual BRMS units have been sequentially deployed at spatially distinct target sites by the capsule robot.

Second, we performed delivery experiments using a custom-designed magnetically actuated capsule robot loaded with four BRMS units. Fig. 5f and g illustrate the locomotion and delivery modes of the capsule robot, respectively. To prevent the BRMS loaded within the capsule channels from mechanical damage and premature loss, an Ecoflex lids assembly was fabricated and integrated into the robot (Fig. S4). The structural components and actuation principles of the capsule robot are detailed in Fig. S3a and S3b. Wireless control of the capsule is achieved via an EMA system composed of six coils, as shown in Fig. 5h. The uniform and gradient magnetic fields generated by this system apply torque and propulsion forces to the embedded permanent magnet, enabling precise 3D navigation to desired positions and orientations. The experimental setup was established by mounting porcine small intestinal tissue within a custom 3D-printed phantom, as depicted in Fig. 5i. Fig. 5j demonstrates the sequential deployment of four BRMS units at the target site using the capsule robot. These results confirm that the capsule robot can stably navigate to the target location under precise external electromagnetic control and accurately release and deploy the BRMS at the designated lesion site. The BRMS delivery process using the magnetically actuated capsule robot is demonstrated in Movie S4.

In conclusion, these results demonstrate the successful and reliable delivery of the proposed BRMS to the GI tract using both a commercial endoscopic system and customized magnetically actuated capsule robots. In particular, the thin, flexible, and rollable form factor of the BRMS enables compatibility not only with the delivery platforms validated in this study but also with other narrow-lumen medical devices, such as feeding tubes and catheters. This high degree of delivery versatility is expected to substantially expand the potential clinical applications of the BRMS.

Supplementary data related to this article can be found online at https://doi.org/10.1016/j.mtbio.2026.103710

The following are the Supplementary data related to this article:

Video 1

Video 2

Video 3

Video 4

### 3.5 *In vivo* test for antitumor efficacy evaluation of BRMS

To evaluate the *in vivo* antitumor efficacy of the developed BRMS, we conducted therapeutic studies in a BALB/c tumor model bearing subcutaneous CT-26 colon carcinoma tumors. The overall treatment timeline is summarized in Fig. 6a. CT-26 cells were subcutaneously inoculated on day −14, and BRMS implantation was performed on day 0 once tumors reached a volume of approximately 85–130 mm^3^. The detailed implantation procedure is described in Fig. S9. From day 0 to day 12, L-arabinose was administered to induce VC1 expression, enabling sustained therapeutic protein secretion from the BRMS. Tumor volume and body weight were monitored throughout the study, and tumors were harvested on day 14 for histological evaluation.

**Fig. 6:**
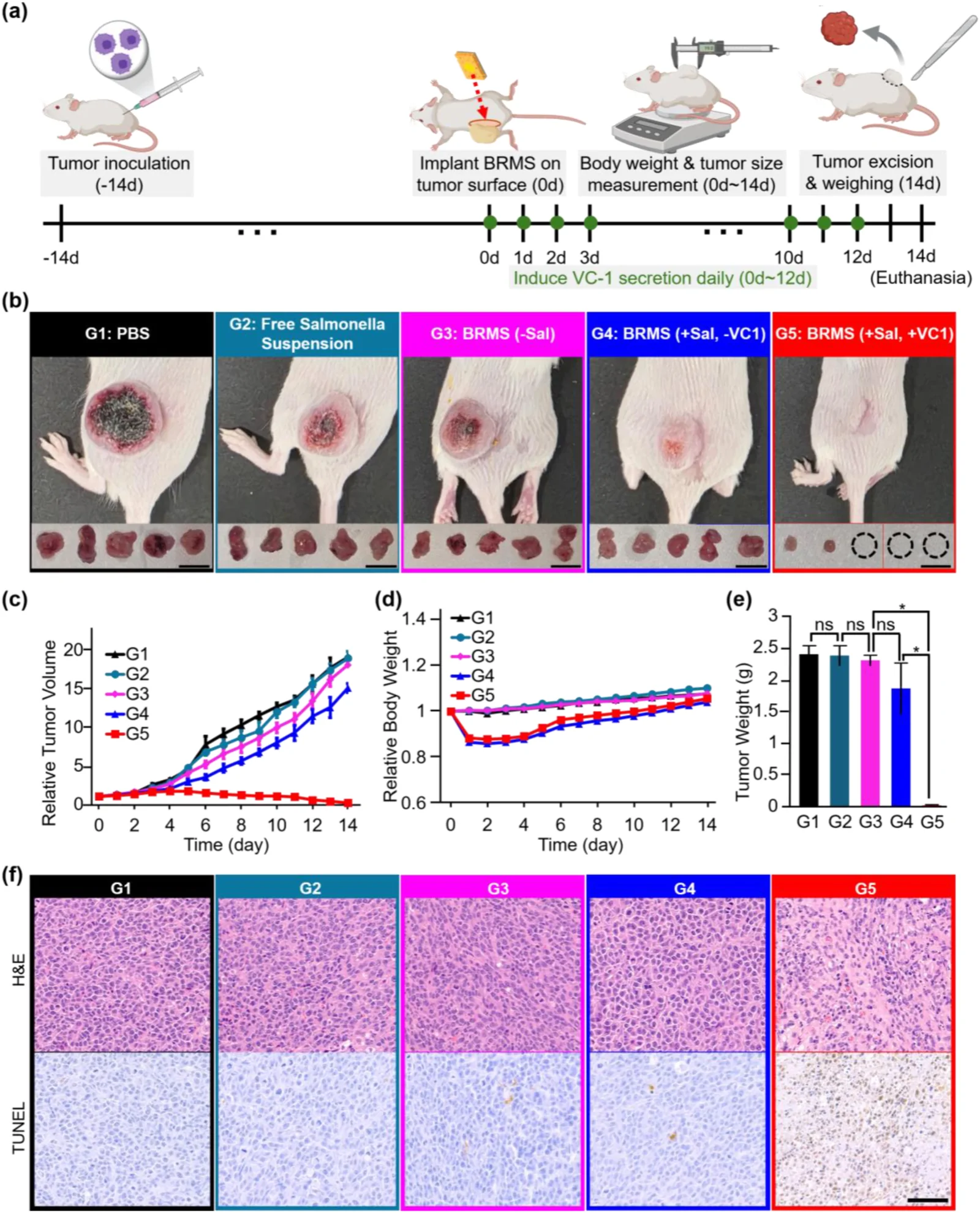
*In vivo* antitumor efficacy of the BRMS in a mouse tumor model. (a) Schematic illustration of the experimental timeline, including subcutaneous CT-26 tumor inoculation, BRMS implantation onto the tumor surface, daily induction of VC1 secretion, and terminal endpoint analysis. (b) Representative photographs of tumor-bearing mice and harvested tumors from each of the five treatment groups (G1–G5) at the experimental endpoint, illustrating the extent of tumor regression (scale bar: 5 mm). (c) Longitudinal tumor growth curves depicting time-dependent changes in tumor volume for each group. (d) Relative body weight monitoring of mice throughout the treatment period to assess systemic toxicity and safety. (e) Quantitative analysis of excised tumor weights at the experimental endpoint. Data are expressed as mean ± s.d.; statistical significance was determined at *p < 0.005. (f) Histopathological evaluation of tumor tissues: hematoxylin and eosin (H&E) staining reveals overall tumor morphology and necrotic regions, while TUNEL staining identifies apoptotic cells within the tumor sections (scale bar: 50 μm).

Mice were randomly allocated to five experimental groups (n = 5 per group): G1 (PBS), control; G2 (Free *Salmonella* suspension), in which an equivalent dose of VC1-secreting *Salmonella* suspension was applied directly onto the tumor surface without the BRMS; G3 (BRMS (−Sal)), BRMS without bacterial loading; G4 (BRMS (+Sal, −VC1)), BRMS loaded with VC1-engineered *Salmonella* without induction of VC1 secretion; and G5 (BRMS (+Sal, +VC1)), BRMS loaded with VC1-engineered *Salmonella* with L-arabinose-induced VC1 secretion. Representative photographs of animals and excised tumors at the study endpoint (day 14) are shown in Fig. 6b. Gross examination revealed large, solid tumors in the G1, G2 and G3 groups, with substantial tumor masses upon excision. The G4 group exhibited moderate tumor growth inhibition relative to controls; however, substantial tumors remained. The G2 group, which received the free *Salmonella* suspension, exhibited tumor growth comparable to that of the control groups throughout the treatment period, without any discernible reduction in tumor size; this outcome is likely attributable to the bacteria being rapidly washed out from the lesion before achieving sufficient penetration into the tumor tissue. In contrast, the G5 group displayed a significant reduction in tumor burden, evident both *in vivo* and after excision, with markedly smaller tumor tissues than all other groups. Longitudinal tumor growth profiles (Fig. 6c; individual tumor trajectories shown in Fig. S10) demonstrated rapid and continuous tumor progression in G1, G2 and G3 throughout the treatment period. Notably, the nearly overlapping growth curves of G1, G2 and G3 indicate that the BRMS polymer matrix is highly biocompatible and does not intrinsically inhibit tumor growth. The G4 group showed an initial delay in tumor expansion followed by tumor regrowth, ultimately approaching control levels, suggesting that bacterial loading, in the absence of therapeutic VC1 expression, is insufficient to achieve durable tumor control in this aggressive CT-26 model. In contrast, G5 exhibited the most pronounced therapeutic response: tumors did not merely show growth suppression, but instead underwent progressive regression, with a sustained reduction in tumor volume over time and a substantial decrease in endpoint tumor size compared with all comparator groups. Notably, the direct comparison between G2 (free *Salmonella* suspension) and G5 (BRMS (+Sal, +VC1))—both receiving the same dose of VC1-secreting *Salmonella*—demonstrates that the therapeutic benefit of the BRMS arises not merely from the bacteria themselves but from the platform's ability to stably retain the bacteria at the target lesion against wash-out, thereby enabling effective tumor penetration and sustained local therapy that the free bacterial suspension, rapidly cleared from the lesion, failed to achieve.

No significant body weight loss or observable signs of systemic toxicity were detected in any treatment group, supporting the favorable *in vivo* safety profile of the BRMS platform and the engineered bacterial component under the tested regimen (Fig. 6d). The excised tumor weights at sacrifice (Fig. 6e) were significantly reduced in G5 compared with the control groups, with only minimal residual tumor mass remaining. Histopathological analyses further substantiated the therapeutic efficacy (Fig. 6f). Hematoxylin and eosin (H&E) staining of tumors from G1, G2, G3 and G4 revealed densely packed, viable tumor tissue with preserved nuclear morphology. In contrast, tumors from G5 exhibited extensive necrotic regions and pronounced apoptotic features. This observation was further supported by TUNEL staining, which showed markedly elevated apoptotic cell populations in G5 relative to all other groups.

Collectively, these findings demonstrate that BRMS-mediated, L-arabinose-inducible VC1 secretion enables robust and localized antitumor efficacy *in vivo*, achieving substantial tumor regression rather than mere growth delay, while maintaining excellent tolerability. These results establish the BRMS as a powerful localized anticancer therapeutic platform for programmed delivery of engineered bacterial therapeutics.

### 3.6 Additional considerations of BRMS

Several technical challenges remain to be addressed for the clinical translation of the BRMS. First, the administration strategy of L-arabinose, the inducer for the VC1 secretion of the engineered *Salmonella* encapsulated within the BRMS, presents a key limitation. In our *in vitro* studies, VC1 induction was achieved exclusively by the L-arabinose co-encapsulated with the bacteria in the central region of the therapeutic layer during fabrication. In contrast, for *in vivo* experiments, L-arabinose was administered via daily intraperitoneal injection to ensure sustained VC1 expression; however, this approach may reduce patient compliance, thereby undermining a major advantage of localized therapy [50,51]. Accordingly, future investigations should focus on enhancing the L-arabinose loading capacity of the BRMS or developing engineered bacterial strains capable of autonomous and sustained VC1 secretion at the target site without external inducers [52]. In addition, the same issue could be addressed by switching the L-arabinose administration route from intraperitoneal injection to oral intake (via drinking water or in capsule form). Oral intake of L-arabinose has been shown not to cause adverse gastrointestinal effects in humans [53,54], and a prior study using a similar arabinose-inducible system achieved a significant antitumor effect by inducing expression at the tumor site through oral supply via drinking water alone [55]; we also plan to examine this option in future work. In this context, it is important to note that excessive protein expression can impose a metabolic burden on the bacteria, potentially compromising their motility and penetration efficiency [56,57]. Consequently, systematic optimization and careful monitoring of VC1 release profiles and bacterial motility will be required to define optimal conditions for therapeutic protein secretion and bacterial loading density.

Second, in our *in vivo* experiments, we demonstrated the therapeutic efficacy of the BRMS against tumor tissue by local implantation in a mouse xenograft model. However, this experimental setup did not fully recapitulate the functional performance of the BRMS within the GI tract. In particular, it did not adequately reflect its capability for local delivery via clinically relevant modalities such as endoscopes or capsule robots, its self-unrolling behavior, or its ability to establish and sustain conformal adhesion with the GI tumor surface, or the real-time kinetics of bacterial activity and VC1 release within the tumor tissue. Therefore, future investigations should focus on orthotopic tumor models in large animals, such as pigs or rabbits, to rigorously evaluate the therapeutic performance of the BRMS in a clinically relevant GI environment.

Finally, biosafety remains a critical consideration for the BRMS translation. Although no significant weight loss or overt adverse effects associated with *Salmonella* administration were observed in the animal experiments, the potential risk of infection cannot be fully excluded, particularly in immunocompromised cancer patients. Accordingly, future research should aim to further enhance the safety profile of the platform by substituting *Salmonella* with clinically validated probiotic bacterial strains as the therapeutic carrier organism [58].

## 4 Conclusion

In this study, we proposed the BRMS, a bacteria-based active drug delivery platform, designed to simultaneously address the major limitations of conventional GI solid tumor therapy: insufficient drug penetration and therapeutic agents wash-out caused by dynamic fluid flow in the GI tract. Through a rationally engineered multilayered architecture, the BRMS integrates stable loading of VC1-secreting *Salmonella*, robust mucoadhesion, and unidirectional therapeutic release into a single platform. This engineering strategy not only protects the therapeutic bacteria and enables stable local retention within the harsh, fluidic GI environment but also creates a localized microenvironment that facilitates effective therapeutic action at the target lesion. Through *ex vivo* experiments using a standard clinical colonoscope and a magnetically actuated capsule robot, we demonstrated the high versatility and translational applicability of the BRMS, enabling precise, non-invasive delivery and deployment to target lesions using existing clinical instruments. In particular, these studies highlight the clinical adaptability of the system for accurate lesion targeting without the need for specialized or invasive procedures. Furthermore, in *in vitro* cancer cell experiments, VC1-secreting *Salmonella* released from the BRMS significantly decreased cancer cell viability compared to freely administered bacteria, confirming that the BRMS-based delivery method effectively enhances local anticancer efficacy. Moreover, in a 3D tumor spheroid model, the BRMS elicited therapeutic effects even within the spheroid core via active bacterial penetration, demonstrating superior intratumoral penetration and enhanced therapeutic potential in solid tumor environments. In *in vivo* studies using a CT-26 colon cancer mouse model, the BRMS induced pronounced tumor regression relative to control groups through the active deep tumor tissue infiltration of the released VC1-secreting *Salmonella*. Importantly, excellent systemic biosafety was observed, with no detectable body weight loss or signs of major organ toxicity throughout the treatment period. In conclusion, the BRMS represents an innovative therapeutic platform that integrates the active therapeutic mechanisms of biological agents with the precise control of engineering delivery systems, offering a potential solution to the limitations of GI therapy. The BRMS proposed in this study is expected to evolve as a next-generation therapeutic strategy with broad applicability to the treatment of various localized diseases, including intractable GI cancers.

## CRediT authorship contribution statement

**Jihun Lee:** Writing – original draft, Validation, Methodology, Investigation, Data curation, Conceptualization. **Sana Ashraf:** Writing – original draft, Investigation, Formal analysis, Data curation. **Eunji Kim:** Methodology, Investigation, Data curation. **Hyo-Jin Lee:** Methodology, Investigation, Data curation. **Joowon Park:** Methodology, Formal analysis. **Heung Jin Jeon:** Supervision, Methodology, Investigation, Data curation. **SeungBeum Suh:** Writing – original draft, Supervision, Investigation, Formal analysis. **Sukho Park:** Writing – review & editing, Validation, Supervision, Investigation, Funding acquisition, Formal analysis, Conceptualization.

## Declaration of competing interest

The authors declare that they have no known competing financial interests or personal relationships that could have appeared to influence the work reported in this paper.

## Data Availability

Data will be made available on request.
