# Supplementary material for: A bacteria-releasing mucoadhesive sheet for localized delivery and penetrative therapy of gastrointestinal cancer
Source: Mater Today Bio. 2026 Sep 23;41:103710. doi: 10.1016/j.mtbio.2026.103710 (PMC13635254; doi:10.1016/j.mtbio.2026.103710)
Supplement: Supplementary file 1 [file mmc1.docx]

**Supporting Information**

**A Bacteria-Releasing Mucoadhesive Sheet for Localized Delivery and Penetrative Therapy of Gastrointestinal Cancer**

*Jihun Lee ^a,1^, Sana Ashraf ^b,c,1^, Eunji Kim ^d^, Hyo-Jin Lee ^d^, Joowon Park ^e^,* *Heung Jin Jeon ^f,*^, SeungBeum Suh ^b,*^, Sukho Park ^a,*^*

^a^ Department of Robotics Engineering, Daegu Gyeongbuk Institute of Science and Technology (DGIST), Daegu 42988, Republic of Korea

^b^ Bionics Research Center, Korea Institute of Science and Technology (KIST), Seoul 02792, Republic of Korea

^c^ Division of Bio-Medical Science & Technology, University of Science and Technology (UST), Daejeon, 34113, Republic of Korea

^d^ Department of Medical Science, College of Medicine, Chungnam National University, Daejeon 35015, Republic of Korea
^e^ School of Electrical and Electronic Engineering, University of Ulsan, Ulsan 44610, Republic of Korea

^f^ Cancer Research Institute, Chungnam National University, Daejeon 34134, Republic of Korea

* Corresponding author. Email address: [livinglogos@cnu.ac.kr](mailto:livinglogos@cnu.ac.kr) (H. Jeon), [keenhurt81@kist.re.kr](mailto:keenhurt81@kist.re.kr) (S. Suh) [shpark12@dgist.ac.kr](mailto:shpark12@dgist.ac.kr) (S. Park)

^1^ These authors contributed equally to this work.

**Table of Supplementary Contents**

Section S1. Image Processing for Quantitation of Signal-Positive Area in Tumor Spheroids

Section S2. Fabrication of BRMS

Section S3. FT-IR Spectroscopy of BRMS and Its Components

Section S4. Operational Principles of the Electromagnetic Actuation System

Section S5. Design and Fabrication of Ecoflex Cap for Endoscopy

Section S6. Design and Fabrication of Ecoflex Lids Assembly for Magnetically Actuated Capsule Robot

**List of Supplementary Figures**

Figure S1. Structural Design and Sequence Information of the FlgM-VC1 Construct.

Figure S2. Design, dimensions, and fabrication of the Ecoflex cap for endoscopic BRMS delivery.

Figure S3. Structural design and magnetic actuation mechanism of the magnetically actuated capsule robot for BRMS delivery.

Figure S4. Design, dimensions, and fabrication of the Ecoflex lids assembly.

Figure S5. Schematic illustration of the BRMS fabrication process.

Figure S6. Quantification of VC1 secreted from VC1-secreting *Salmonella*.

Figure S7. Cytotoxicity assessment of VC1 secreted from engineered *Salmonella* in colon cancer cell lines.

Figure S8. *Ex vivo* demonstration of endoscopic BRMS delivery using a porcine small intestine phantom.

Figure S9. *In vivo* implantation procedure of the BRMS onto the tumor site.

Figure S10. Individual tumor growth profiles of mice across treatment groups.

**List of Supplementary Tables**

Supplementary Table 1. *Salmonella* strains.

Supplementary Table 2. Primers used in this study.

**List of Supplementary Movies**

Movie S1. Unrolling of BRMS on a porcine small intestine

Movie S2. *Ex vivo* BRMS delivery test using endoscopy on an opened porcine small intestine

Movie S3. *Ex vivo* BRMS luminal delivery into an intact porcine small intestine

Movie S4. *Ex vivo* BRMS delivery test using magnetically actuated capsule robot

**S1. Image Processing for Quantitation of Signal-Positive Area in Tumor Spheroids**

To quantify the radial distribution profile of the signal-positive area in CT-26 tumor spheroid images acquired with a confocal laser scanning microscope, we developed a custom Python-based image processing routine. This algorithm consists of three components. (i) Spheroid boundary segmentation and radial coordinate normalization: using the DAPI channel image, the spheroid boundary was identified by intensity-based thresholding, and each pixel was assigned a normalized radial position (0 = surface, 1 = center) based on the local boundary radius measured from the centroid of the mask. This approach can be accurately applied even when the spheroid periphery is not a perfect circle. (ii) Conversion of grayscale fluorescence intensity images to binary images: each pixel was classified as signal-positive or signal-negative using an intensity threshold determined independently for each fluorescence channel, based on the background-subtracted pixel intensity distribution within the spheroid. (iii) Construction of the radial distribution profile: using the radial coordinates from (i) and the binary images from (ii), the percentage (%) of pixels classified as signal-positive within the corresponding ring area was calculated at multiple positions along the normalized radius.

**S2. Fabrication of BRMS**

The BRMS consists of a three-layer structure—a guard layer, an unrolling layer, and a therapeutic layer—and was fabricated by adapting a method reported in a previous study [1-3]. First, as shown in Figure S5a, a two-layer structure composed of the guard and unrolling layers was fabricated using UV photopolymerization. The solutions of TEGDM (Mw 286.3, Merck) and PEGDM (Mw 750, Merck) were prepared by mixing a photoinitiator, 2-hydroxy-2-methylpropiophenone, into each at a concentration of 1% (v/v). To fabricate the guard layer, the TEGDM solution was dispensed onto a cover glass, covered with a silane-coated cover glass, and subsequently exposed to UV irradiation (365 nm, 230 mW/cm^2^) for 60 seconds. After removal of the cover glass, the PEGDM solution was dispensed onto the cured guard layer. The assembly was again covered with a cover glass and subjected to UV irradiation (365 nm, 230 mW/cm^2^) for 30 seconds. Finally, the slide glass was removed, yielding the two-layer structure consisting of the guard and unrolling layers.

Next, a therapeutic layer was constructed on the surface of the guard layer of the two-layer structure (Figure S5b). In this study, a sequential casting process using Ecoflex molds was used to fabricate the bacteria-loading central region and the mucoadhesive peripheral region of the therapeutic layer. First, master molds for the central and peripheral regions of the therapeutic layer were fabricated using an SLA 3D printer (Form4, Formlabs). Ecoflex 00-30 (Smooth-On, USA) was poured onto the printed master molds and cured to produce negative Ecoflex replica molds for subsequent casting. To fabricate the therapeutic layer, a 1 wt% sodium alginate solution (Junsei, Japan) was sterilized by autoclaving, and a 10 wt% skim milk solution (MBcell, South Korea) was prepared. First, the Ecoflex mold defining the central region of the therapeutic layer was placed on the guard layer, and a mixture of 10 wt% skim milk solution containing 10^7^ CFU of *Salmonella* and 20 wt% L-arabinose (Mw 150.13, Merck) at a 10:1 volume ratio was cast into the mold. Subsequently, the Ecoflex mold defining the peripheral region of the therapeutic layer was positioned, and a mucoadhesive solution composed of 10 wt% skim milk and 1 wt% alginate mixed at a 1:5 (v/v) ratio was cast, followed by freezing at -80°C. After freeze-drying, the excess area of the two-layer structure was trimmed to obtain the final flat-shaped BRMS. Finally, rolling and thermal treatment processes were performed to enable the delivery of the BRMS using endoscopic tools or a capsule robot (Figure S5c). Using tweezers, the BRMS was rolled into a cylindrical configuration with the therapeutic layer facing outward and then inserted into a custom 3D-printed cylindrical resin jig to maintain the rolled geometry. To stabilize the rolled configuration, thermal treatment was applied to the BRMS inside the jig using a heating gun at 120°C for 5 seconds. This heat treatment fixes the rolled shape while preserving the physical properties required for subsequent self-unrolling. The completed rolled BRMS can be reliably loaded into the working channel of a clinical endoscope or the delivery channel of a magnetically actuated capsule robot.

**S3. FT-IR spectroscopy of BRMS and Its Components**

The chemical compositions of the BRMS and its individual constituent layers were analyzed using FT-IR spectroscopy (Figure 2g). First, we examined the spectrum of the unrolling layer, which is composed of PEGDM. In this FT-IR spectrum, characteristic absorption peaks corresponding to methyl groups, carbonyl groups (C=O), and aliphatic C–O groups were observed at 2870, 1735, and 1060–1030 cm^-1^, respectively [4]. Second, for the guard layer composed of TEGDM, peaks corresponding to C=C bonds, methylene groups, and ether groups were identified at 1635, 1452, and 1000–1200 cm^-1^, respectively [5]. Finally, the therapeutic layer was analyzed. This layer consists of a peripheral region (alginate, skim milk) and a central region (skim milk, *Salmonella*, L-arabinose). In the FT-IR spectrum of the peripheral region, the characteristic peaks of sodium alginate—specifically, the asymmetric and symmetric stretching vibrations of carboxylate groups—were identified near 1650 cm^-1^ and 1460 cm^-1^, respectively, while the C-O stretching of the pyranosyl ring was observed near 1030 cm^-1^ [6]. Additionally, the Amide I band (C=O stretching, ~1657 cm^-1^) and Amide II (N-H bending and C-N stretching, ~1549 cm^-1^) band, which are characteristic of peptide bonds in skim milk, were observed to overlap with the alginate peaks, confirming the formation of a composite between alginate and skim milk [7]. In the spectrum of the central region, Amide I and Amide II bands derived from skim milk proteins were predominantly observed. Although the specific absorbance wavenumbers for L-arabinose have been reported at 1052 cm^-1^ and 979 cm^-1^ in the literature, they appeared as a complex band in the 1000–1100 cm^-1^ region in this experiment due to spectral overlap with other components, such as lactose (~1074 cm^-1^) present in skim milk [8]. Finally, the FT-IR spectrum of the entire BRMS was confirmed to contain all the major characteristic peaks of the individual layers, indicating that the polymeric components were successfully laminated without chemical degradation or denaturation during the fabrication process.

**S4. Operational Principles of the Electromagnetic Actuation System**

The capsule robot proposed in this study is actively controlled using precise modulated magnetic fields generated by an electromagnetic actuation (EMA) system composed of six coils (Figure 5h). The locomotion strategy of the capsule involves two sequential stages: orientation alignment and propulsion. First, a uniform magnetic field is applied to align the capsule in the desired direction, followed by the generation of a gradient magnetic field to propel the capsule toward the target location. The magnetic rotational torque ($\boldsymbol{\tau}_{\mathbf{B}}$) exerted on the capsule within the uniform magnetic field is determined by the interaction among the magnetization (**M**) and volume (**V**) of the permanent magnet embedded within the capsule, and the magnetic flux density (**B**) generated by the EMA system.

$$\boldsymbol{\tau}_{\mathbf{B}}=V\left( \boldsymbol{M\times B} \right)$$

(1)

In particular, the permanent magnet assembly that translates along the center rod of the capsule is designed to possess a diagonally oriented magnetization vector. Due to this feature, the capsule can be precisely aligned in a desired three-dimensional direction by applying a slightly tilted uniform magnetic field. The magnetic field at a specific point **P** (x, y, z) is generated by the input currents supplied to each coil of the 6-coil EMA system. The resultant magnetic flux density at point **P**, denoted as $\mathbf{B(P)}$, is expressed as a linear relationship between the mapping matrix ($\hat{\mathbf{b}}\left( \mathbf{P} \right)$), which represents the magnetic field per unit current, and the input current vector (**I**) applied to the coils.

$$\mathbf{B}\left( \mathbf{P} \right)\mathbf{=}\hat{\mathbf{b}}\left( \mathbf{P} \right)\mathbf{I}$$

(2)

Furthermore, the magnetic force (**F_magnet_**) exerted on the capsule by the gradient magnetic field is a key factor in generating propulsion. Consequently, the magnetic field (**B**) and magnetic force (**F**) generated by the EMA system can be integrated and expressed as a linear relationship between the mapping matrix and the input current.

$$\mathbf{F}_{\mathbf{magnet}}=V\left( \boldsymbol{M\cdot\nabla} \right)\mathbf{B}$$

(3)

Thus, the magnetic field and magnetic force generated by the currents flowing through the EMA system can be expressed using the mapping matrix **X(P)** as follows:

$$\left[ \begin{matrix} \mathbf{B} \\ \mathbf{F} \end{matrix} \right]\mathbf{=}\left[ \begin{matrix} \hat{\mathbf{b}}\left( \mathbf{P} \right) \\ V\left( \boldsymbol{M\cdot\nabla} \right)\hat{\mathbf{b}}\left( \mathbf{P} \right) \end{matrix} \right]\mathbf{I=X}\left( \mathbf{P} \right)\mathbf{I}$$

(4)

To control the capsule, the input currents required for each coil to exert a specific magnetic field and force on the permanent magnet can be calculated in real-time using the pseudoinverse of the mapping matrix (${\mathbf{X}\left( \mathbf{P} \right)}^{\mathbf{+}}$).

$$\mathbf{I=}{\mathbf{X}\left( \mathbf{P} \right)}^{\mathbf{+}}\left[ \begin{matrix} \mathbf{B} \\ \mathbf{F} \end{matrix} \right]_{\mathbf{desired}}$$

(5)

**S5. Design and Fabrication of Ecoflex Cap for Endoscopy**

In the endoscope-assisted BRMS delivery system, an Ecoflex cap is mounted at the distal tip of the endoscope. Its primary role is to protect the BRMS loaded inside the working channel from damage caused by the influx of gastrointestinal (GI) fluid ingress and to prevent the unintentional BRMS loss during endoscopic navigation. The Ecoflex cap was designed in an initially unfolded state, as shown in Figure S2a, with dimensions and geometry precisely matched to physically seal the working channel inlet. During endoscopic navigation within the GI tract, the cap remains in the unfolded state, thereby effectively sealing the working channel inlet. Upon reaching the target lesion site, when the forceps inserted through the working channel push the BRMS, the applied mechanical force induces a transition of the cap into a folded, open state, enabling controlled deployment of the BRMS (Figure S2b). After the BRMS deployment, the cap is designed to elastically recover to its initial unfolded configuration by leveraging the inherent elasticity of the Ecoflex material. The fabrication process of the Ecoflex cap was carried out using a molding process, as illustrated in Figure S2d. First, a resin mold featuring the negative shape of the cap was fabricated using an SLA 3D printer (Form 4, Formlabs). To facilitate the demolding of the Ecoflex, the fabricated mold was surface-treated with silane (trichloro(1H,1H,2H,2H-perfluorooctyl) silane, Merck) at 80°C for 1 hour. Subsequently, Ecoflex 00–30 (Smooth-On Inc., USA) was cast into the mold, and excess material was removed and planarized using a razor blade. After thermal curing at 80°C for 2 hours, the cured elastomer was carefully detached from the mold to obtain the final Ecoflex cap. The photograph of the fabricated Ecoflex cap is shown in Figure S2c.

**S6. Design and Fabrication of Ecoflex lids assembly for Magnetically Actuated Capsule Robot**

The BRMS loaded within the capsule robot proposed in this study must be effectively protected from the harsh GI environment by the Ecoflex lids assembly and safely transported to the target lesion site. The Ecoflex lids assembly is designed to include a central opening for integration with the capsule body and four individual Ecoflex lids that seal each capsule channel (Figure S4a). The assembly is composed of a disk-shaped base component matching the diameter of the capsule channel (diameter: 3.5 mm, height: 0.5 mm) and a secondary disk component with a slightly larger diameter (diameter: 3.7 mm, height: 0.5 mm) to completely cover and seal the channel opening. When the internal pushing bar, actuated via magnetic control, advances to expel the BRMS from a specific channel, the corresponding Ecoflex lid at the channel inlet elastically folds and opens, allowing controlled deployment of the BRMS (Figure S4b). After the BRMS is fully deployed, the folded lid returns to its original closed configuration due to the inherent elasticity of the Ecoflex material, thereby re-sealing the channel. The Ecoflex lids assembly was fabricated using a molding-based process, as illustrated in Figure S4d. First, a resin mold was fabricated using an SLA 3D printer (Form 4, Formlabs), followed by surface silanization with trichloro(1H,1H,2H,2H-perfluorooctyl) silane (Merck) at 80°C for 1 hour. Subsequently, Ecoflex 00–30 (Smooth-On Inc., USA) was poured into the mold, and excess Ecoflex solution was removed and planarized using a razor blade. After thermal curing at 80°C for 2 hours, the cured Ecoflex part was carefully detached from the mold to obtain the final Ecoflex lids assembly. The photograph of the fabricated Ecoflex lids assembly is shown in Figure S4c.

**Figure S1. Structural design and sequence information of the FlgM-VC1 construct.** (a) Schematic diagram of the FlgM-VC1 fusion construct. (b) Amino acid sequence of FlgM-VC1. (c) Nucleotide sequence of *flgM-VC1*.

**Figure S2. Design, dimensions, and fabrication of the Ecoflex cap for endoscopic BRMS delivery.** (a) Schematic illustration of the structure and dimensions of the Ecoflex cap in the unfolded (closed) configuration, designed to hermetically seal the endoscopic working channel and prevent premature hydration of the BRMS during endoscopic navigation. (b) Schematic representation of the Ecoflex cap in the folded (open) configuration, illustrating the elastic deformation that enables on-demand deployment of the rolled BRMS upon mechanical actuation by endoscopic forceps. (c) Representative photograph of the fabricated Ecoflex cap (scale bar: 10 mm). (d) Schematic overview of the Ecoflex cap fabrication process.

**Figure S3. Structural design and magnetic actuation mechanism of the magnetically actuated capsule robot for BRMS delivery.** (a) Exploded-view schematic of the capsule robot, illustrating the Ecoflex lids assembly, four rolled BRMS units, capsule body, center rod, magnetic driving unit, and capsule head (scale bar: 10 mm). (b) Sequential photographic images with corresponding schematic illustrations of the capsule under external magnetic actuation, demonstrating BRMS deployment from the capsule.

**Figure S4. Design, dimensions, and fabrication of the Ecoflex lids assembly.** (a) Schematic illustration of the structure and dimensions of the Ecoflex lids assembly in the unfolded (closed) configuration, designed to seal the capsule channel opening and protect the loaded BRMS from the surrounding fluids during capsule locomotion. (b) Schematic representation of the Ecoflex lids assembly in the folded (open) configuration, illustrating the elastic deformation of the Ecoflex lids assembly that enables on-demand opening for controlled BRMS deployment. (c) Representative photograph of the fabricated Ecoflex lids assembly (scale bar: 5 mm). (d) Schematic overview of the mold-assisted fabrication process of the Ecoflex lids assembly.

**Figure S5. Schematic illustration of the BRMS fabrication process.** (a) Stepwise construction of the 2-layer structure by sequential UV photopolymerization of the unrolling layer, followed by the guard layer. (b) Formation of the therapeutic layer on top of the 2-layer structure, involving mold-assisted casting of a bacteria-loaded central region and a mucoadhesive peripheral region, followed by freeze-drying to establish the final functional architecture. (c) Preparation of the rolled BRMS is achieved by mechanically rolling the planar BRMS within a cylindrical resin jig, followed by thermal fixation prior to loading into the endoscopic delivery channel.

**Figure S6. Quantification of VC1 secreted from VC1-secreting *Salmonella*.** VC1 secretion was induced with L-arabinose for 6 h, and the amount of VC1 in the culture supernatant was quantified using a His-tag ELISA.

**Figure S7. Cytotoxicity assessment of VC1 secreted from engineered *Salmonella* in colon cancer cell lines.** (a) Representative microscopic images of colon cancer cell lines treated with concentrated bacterial culture supernatant for 48 hours (x100 magnification). Scale bar: 300 μm. (b) Quantitative evaluation of cell viability in colon cancer cell lines measured using the WST-8 assay 24 hours after treatment with concentrated culture supernatants. Data are presented as mean ± s.d.; statistical significance was determined at **p < 0.0005 and ***p < 0.0001.

**Figure S8. *Ex vivo* demonstration of endoscopic BRMS delivery using a porcine small intestine phantom.** (a) Photograph of the clinical endoscopic system utilized in the *ex vivo* BRMS delivery study, illustrating the endoscope, imaging unit, and configuration of the working channel. (b) Top-view photograph of an intact porcine small intestine positioned inside a custom 3D-printed intestine phantom before endoscopic insertion, demonstrating the intraluminal delivery setup without surgical incision. (c) Representative endoscopic images acquired during sequential BRMS deployment, showing the immediate post-release configuration followed by hydration-induced self-unrolling of the BRMS within the porcine small intestine.

**Figure S9. *In vivo* implantation procedure of the BRMS onto the tumor site.** Schematic illustrations (top) and corresponding photographic images (bottom) depicting the surgical implantation procedure of the BRMS in a CT-26 tumor–bearing mouse model. Following surgical incision of the subcutaneous tumor, the BRMS was carefully positioned on the tumor surface, after which the skin incision was sutured and closed.

**Figure S10. Individual tumor growth profiles of mice across treatment groups.** (a–e) Time-dependent changes in tumor volume for individual mice (n = 5 per group) in each experimental group: (a) G1 (PBS control), (b) G2 (free *Salmonella* suspension), (c) G3 [BRMS (−Sal)], (d) G4 [BRMS (+Sal, −VC1)], and (e) G5 [BRMS (+Sal, +VC1)]. Each curve corresponds to the tumor growth profile of a single mouse throughout the treatment period.

**Supplementary Table 1.** *Salmonella* strains.

| **Number** | **Name** | **Relative genotype & information** | **Used** |
| --- | --- | --- | --- |
| **#26** | *Salmonella* enterica subsp. enterica serovar Typhimurium str. LT2 | Taxonomy ID: 99287 (ATCC 700720) | Lab strain |
| **#46** | SF586 | Transformation host when transferring from *E. coli* to *Salmonella* | From Professor In soo Lee at Hannam University |
| **#177** | BRD509 asd *S. typhimurium* | Mutant aroA/aroD/asd variant of *Salmonella* strain SL1344 | From Professor In soo Lee at Hannam University |
| **#178** | *S. typhimurium* BRD509 | Mutant aroA/aroD variant of *Salmonella* strain SL1344 | From Professor In soo Lee at Hannam University |
| **#245** | *S. typhimurium* BRD509 rcsB | Mutant aroA/aroD/rcsB variant of *Salmonella* strain SL1344 | In this study |
| **#1237** | ST: BRD509 asd rcsB *S. typhimurium* | Mutant aroA/aroD/asd/rcsB variant of *Salmonella* strain SL1344 | In this study |
| **#1423** | flgM-VC1-fhlDC-pBAD18-asd+ / #1237 | flgM-VC1-fhlDC-pBAD18-asd+ / #1237 (aroA aroD rcsB asd −) | In this study |

**Supplementary Table 2.** Primers used in this study.

| **Primer name** | **Sequence** |
| --- | --- |
| **flgM-NheI-For** | GATCGATCGCTAGCAGGAGGTTTGACCTATGAGCATTGACCGTACCT |
| **VC1-SacI-Re** | GATCGATCGAGCTCTTAGCAAATTTCCGGATGATCATAGTTGCAGCG |
| **rcsB-pkd13-For** | ATCAGCGACATTGACGCCTACGTCAAAAGCTTGCTGCTAGCGTGTAGGCTGGAGCTGCTTCGAAGTT |
| **rcsB-pkd13-Re** | CCTGATAAGCGTAGCGCCATCAGGCTGGGTAACATAAAAGCTGTCAAACATGAGAATTAATTCGGGGA |
| **flhD-SacI-For** | TCGATCGAGCTCAGGAGGTTTGATCCTATGGGAACAATGCATACATCCGAGTTGCT |
| **flhC-SacI-Re** | TCGATCGTCGACTTAAACAGCCTGTTCGATCTGTTCATCCAGCAGTT |
| **asd-ClaI-For** | GGTGCATCGATCAGGAAAAAACGCTATGAAAAATGTTGGT |
| **asd-ClaI-Re** | TCGATCATCGATCTACGCCAACTGGCGCAGCATTCGACGCAGCG |

**References**

[1]	J. Lee, S. Park, D. Yoon, C. Murugan, S. Bang, S. Park, Targeted drug delivery using self-unrolling sheets in magnetically actuated capsules, Device 3 (2025) 100654. <https://doi.org/10.1016/j.device.2024.100654>.

[2]	T. Kondo, Z.K. Nezhad, J. Suzuki, N. Nagai, M. Nishizawa, T. Abe, H. Kaji, A self-deploying drug release device using polymeric films, J. Biomed. Mater. Res. B Appl. Biomater. 106 (2018) 780 – 786. <https://doi.org/10.1002/jbm.b.33887>.

[3]	Y. Sato, N. Nagai, T. Abe, H. Kaji, A multilayered sheet-type device capable of sustained drug release and deployment control, Biomed. Microdevices 21 (2019) 60. <https://doi.org/10.1007/s10544-019-0411-z>.

[4]	J.A. Mathew, V. Kache, C. Liu, L. Tang, J. Yang, Nano-featured highly interconnective macroporous elastic scaffolds for cardiovascular tissue engineering, J. Biomed. Mater. Res. A 100A (2012) 1647 – 1654. <https://doi.org/10.1109/EMBSW.2007.4454169>.

[5]	L. Hodásová, C. Alemán, L.J. del Valle, L. Llanes, G. Fargas, E. Armelin, 3D-Printed polymer-infiltrated ceramic network with biocompatible adhesive to potentiate dental implant applications, Materials 14 (2021) 5513. <https://doi.org/10.3390/ma14195513>.

[6]	H. Daemi, M. Barikani, Synthesis and characterization of calcium alginate nanoparticles, sodium homopolymannuronate salt and its calcium nanoparticles, Sci. Iran. 19 (2012) 2023 – 2028. <https://doi.org/10.1016/j.scient.2012.10.005>.

[7]	A. Rachah, O. Reksen, V. Tafintseva, F.J.M. Stehr, E.O. Rukke, E. Prestløkken, A. Martin, A. Kohler, N.K. Afseth, Exploring dry-film FTIR spectroscopy to characterize milk composition and subclinical ketosis throughout a cow's lactation, Foods 10 (2021) 2033. <https://doi.org/10.3390/foods10092033>.

[8]	M. Černá, A.S. Barros, A. Nunes, S.M. Rocha, I. Delgadillo, J. Čopíková, M.A. Coimbra, Use of FT-IR spectroscopy as a tool for the analysis of polysaccharide food additives, Carbohydr. Polym. 51 (2003) 383 – 389. <https://doi.org/10.1016/S0144-8617(02)00259-X>.
